# Systematic review and meta-analysis of preclinical studies testing mesenchymal stromal cells for traumatic brain injury

**DOI:** 10.1038/s41536-021-00182-8

**Published:** 2021-10-29

**Authors:** Francesca Pischiutta, Enrico Caruso, Alessandra Lugo, Helena Cavaleiro, Nino Stocchetti, Giuseppe Citerio, António Salgado, Silvano Gallus, Elisa R. Zanier

**Affiliations:** 1 Laboratory of Acute Brain Injury and Therapeutic Strategies, Department of Neuroscience, Istituto di Ricerche Farmacologiche Mario Negri IRCCS, Milan, Italy; 2 Neuroscience Intensive Care Unit, Department of Anesthesia and Critical Care, Fondazione IRCCS Cà Granda Ospedale Maggiore Policlinico, Milan, Italy; 3 Laboratory of Lifestyle Epidemiology, Department of Environmental Health Sciences, Istituto di Ricerche Farmacologiche Mario Negri IRCCS, Milan, Italy; 4 Life and Health Sciences Research Institute (ICVS), School of Medicine, University of Minho, Braga, Portugal; 5 ICVS/3B’s—PT Government Associate Laboratory, Braga/Guimarães, Portugal; 6 Stemmatters, Biotechnology and Regenerative Medicine, Guimarães, Portugal; 7 Department of Pathophysiology and Transplants, University of Milan, Milan, Italy; 8 School of Medicine and Surgery, University of Milano-Bicocca, Milan, Italy

**Keywords:** Brain injuries, Mesenchymal stem cells

## Abstract

Mesenchymal stromal cells (MSCs) are widely used in preclinical models of traumatic brain injury (TBI). Results are promising in terms of neurological improvement but are hampered by wide variability in treatment responses. We made a systematic review and meta-analysis: (1) to assess the quality of evidence for MSC treatment in TBI rodent models; (2) to determine the effect size of MSCs on sensorimotor function, cognitive function, and anatomical damage; (3) to identify MSC-related and protocol-related variables associated with greater efficacy; (4) to understand whether MSC manipulations boost therapeutic efficacy. The meta-analysis included 80 studies. After TBI, MSCs improved sensorimotor and cognitive deficits and reduced anatomical damage. Stratified meta-analysis on sensorimotor outcome showed similar efficacy for different MSC sources and for syngeneic or xenogenic transplants. Efficacy was greater when MSCs were delivered in the first-week post-injury, and when implanted directly into the lesion cavity. The greatest effect size was for cells embedded in matrices or for MSC-derivatives. MSC therapy is effective in preclinical TBI models, improving sensorimotor, cognitive, and anatomical outcomes, with large effect sizes. These findings support clinical studies in TBI.

## Introduction

Every year, worldwide, about 69 million people suffer traumatic brain injury (TBI), about 13 million of them moderate to severe^1^. Survivors often experience severe and persistent motor and cognitive deficits, resulting in a huge economic burden on society^2–4^. Although major improvements have been achieved in patient care, with an overall reduction of mortality^5,6^, no neuroprotective or restorative therapies are yet available.

Mesenchymal stromal cells (MSCs) are multipotent progenitor cells first isolated from bone marrow^7^, and subsequently from many other sources including adipose tissue and birth-related tissues (umbilical cord and umbilical cord blood, amniotic fluid, or placenta)^8^. MSCs are attractive candidates for cell therapy because of their ease of isolation and ex vivo expansion, their low immunogenicity, and high immunosuppressive activity^9^.

Twenty years ago the first experimental study assessing MSC protective effects in TBI was published^10^. Since then interest has grown, attracting many research groups worldwide^11–13^. Results are promising but are hampered by the wide variability of therapeutic effects. Besides conceptual issues and methodological differences between laboratories regarding the TBI models (species and strain used, sex, age, anesthetic agent, TBI model, etc.), and the administration protocol (dose, delivery route, and time of MSC infusion), the heterogeneity of MSC populations related to their tissue of origin can also affect the outcome. Other elements contributing to heterogeneity are represented by MSC modifications introduced to boost efficacy including the use of matrices^14,15^, genetic manipulations^16,17^, or in vitro pre-conditioning^18^. More recently, cell-free approaches based on MSC bioactive factors (secretome or extracellular vesicles) have been tested^19^.

Although MSC-based approaches have proved beneficial in TBI models, the optimal protocol, manipulation, or cell product is still debated. Systematic reviews and meta-analyses of preclinical studies can offer a rational and sensitive method to grade preclinical evidence, with the aim of proceeding to clinical application. A systematic review and meta-analysis of MSC effects on locomotor recovery in TBI models were published by Peng et al.^20^. Since then publications in this field have almost doubled. Here we aim at updating and integrating the previous work, assessing MSC effects on sensorimotor and cognitive functions and anatomical damage after experimental TBI. We also examined the efficacy of MSCs in relation to measures of clinical interest such as their source and type of transplant, timing, dose, and route of administration as well as model-related variables (species, sex, and TBI models). Last, we analyzed the effects of MSC manipulations as potential boosters of efficacy.

## Results

### Study selection and characteristics

**Fig. 1:**
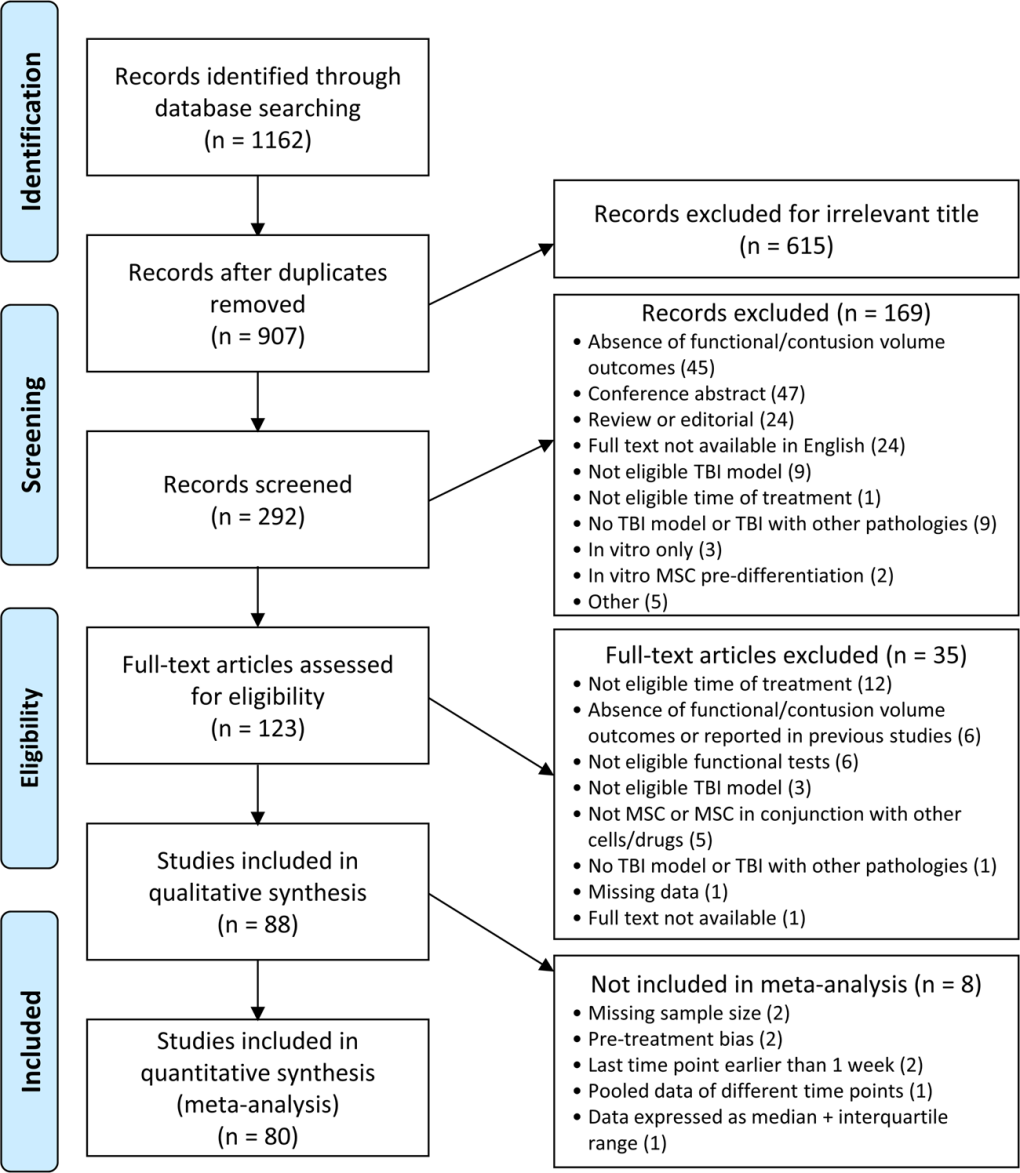
PRISMA flow diagram of the studies. Flow chart represents the selection process. A total of 80 studies were included in the meta-analysis.

Our database inquiry aimed at assessing the preclinical evidence of MSC efficacy on sensorimotor, cognitive, and anatomical outcomes after TBI identified 1162 results, of which 88^9,10,14–18,21–101^ met the eligibility criteria and are reported in the narrative descriptions and in Supplementary Table 1, though only 80 were included in the meta-analysis, as detailed in Fig. 1.

**Fig. 2:**
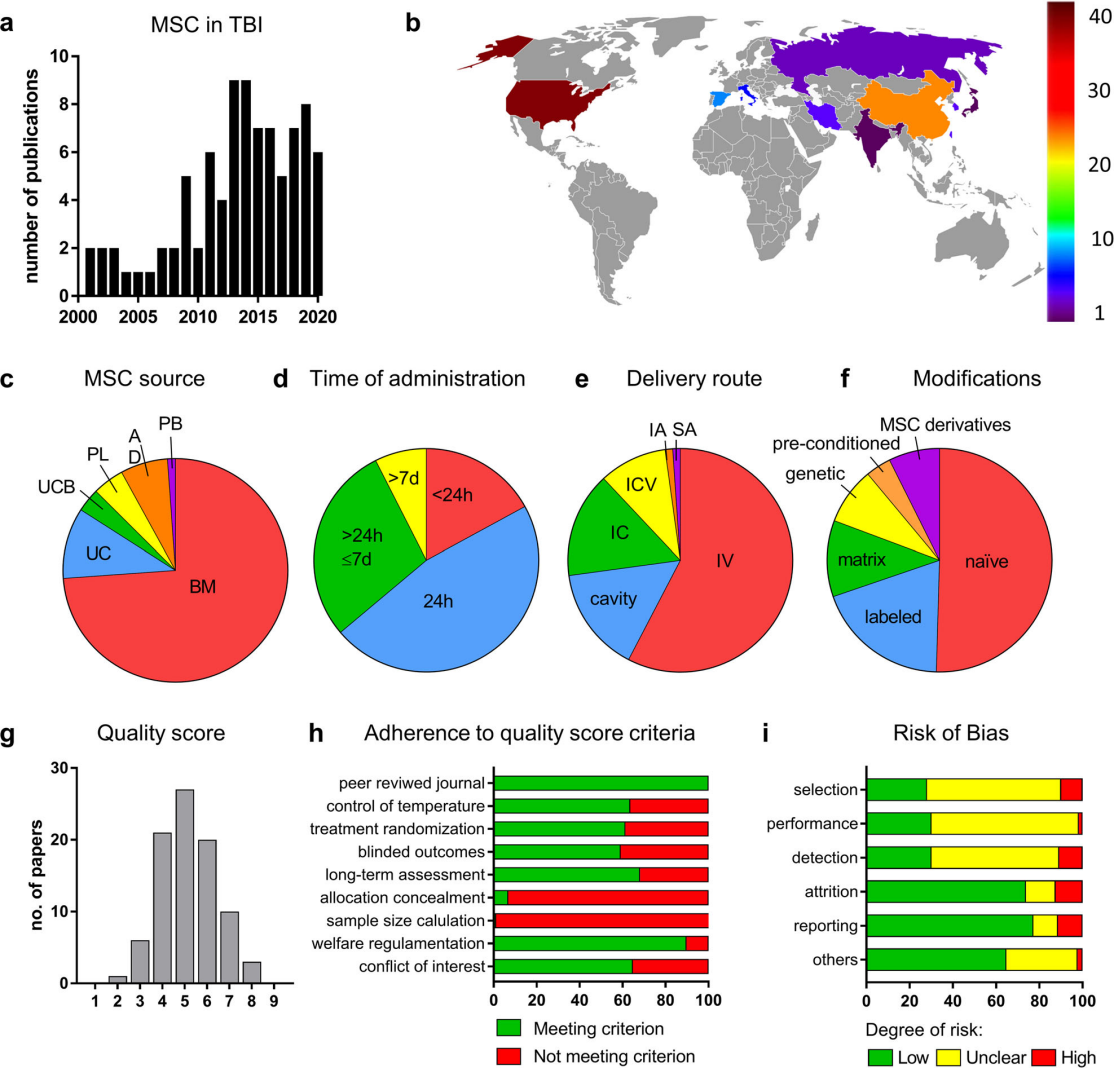
Characteristics of the 88 studies in the qualitative synthesis, quality score, and risk of bias. **a** Number of publications per year. **b** World map with a color scale indicating the number of papers published in each country (image adapted from Freepik.com). **c**–**f** Pie charts of features of publications related to MSC source (**c**), time of administration (**d**), delivery route (**e**), and modifications (**f**). **g** Distribution of quality scores. **h** Percentages of studies meeting each quality score criterion. **i** Risk of bias: percentages of low risk (green), unclear risk (yellow), and high risk (red) for each category.

The first study was published in 2001 and the rate of publications/year then increased (2020 was screened up to June) (Fig. 2a). Across countries, the largest contribution came from the USA (with 38 studies) and China (26), followed by Spain (6), Italy (4), South Korea (4), Iran (3), Taiwan (3), Russia (2), Japan (1), and India (1) (Fig. 2b). Rats, mainly Wistar (*n* = 34) or Sprague Dawley (*n* = 32) were used in most of the studies (*n* = 66, 75% of papers), mice in the remainder (*n* = 17, 19.3% of papers) (Supplementary Fig. 1a). Rodents were predominantly male (*n* = 61, 69.3% of papers) (Supplementary Fig. 1b). Controlled cortical impact (CCI, *n* = 55) and weight drop (WD, *n* = 25) impact injuries were the most frequently used TBI models (62.5% and 28.4% of papers) (Supplementary Fig. 1c). Among anesthetics, chloral hydrate (*n* = 41) and isoflurane (*n* = 17) were the most often used (46.6% and 19.3% of papers). Only 6 studies (6.8%) used an immunosuppressant (cyclosporine A); 5 studies (5.7%) used antibiotics (penicillin) and 14 (15.9%) analgesics (8 buprenorphine, 4 morphine/meloxicam, 2 ketoprofen, 1 tramadol, 1 acetaminophen, 1 bupivacaine, 1 not specified).

MSCs were mostly from bone marrow (BM, *n* = 65, 73.9% of papers), followed by umbilical cord (UC, *n* = 9, 10.2%), adipose tissue (AD, *n* = 6, 6.8%), placenta (PL, *n* = 4, 4.5%), umbilical cord blood (UCB, *n* = 3, 3.4%), and peripheral blood (PB, *n* = 1, 1.1%) (Fig. 2c). The median time between TBI and MSC delivery was 24 h (*n* = 44, 50% of papers) (Fig. 2d). Only 4 papers (4.5%) tested multiple doses. The most frequent delivery route was intravenous (IV) (*n* = 53, 60.2% of papers) (Fig. 2e). As regards MSC modifications, 55 studies (62.5% of papers) presented at least one arm with naïve MSCs, 21 (23.8%) with labeled MSCs, 12 (13.6%) with matrix-embedded MSCs, 9 (10.2%) with genetically modified MSCs, 4 (4.5%) with MSCs subjected to in vitro pre-conditioning before transplant and 8 (9.1%) with MSC derivatives (either exosomes or secretome, Fig. 2f).

### Quality score and risk of bias

Quality score was determined with the checklist modified from the Collaborative Approach to Meta-Analysis and Review of Animal Data from Experimental Studies (CAMARADES) (Supplementary Table 2 for complete evaluation, Fig. 2g, h for summary data). The median quality score across the 88 studies was 5 (interquartile range 4–6, range 2–8). Most studies were of moderate to high quality (moderate [scores 4–6]: 68 studies; high [scores 7–9]: 13 studies). The percentage of adherence for each quality score criterion showed that very few studies reported allocation concealment of experimental groups (6.8%) and sample size calculation (1.1%) (Fig. 2h).

According to SYRCLE’s RoB tool, all the studies were considered at moderate to high risk of bias (RoB) (Supplementary Table 3 for complete evaluation, Fig. 2i for summary data). RoB assessment revealed a low risk of 42.0%, unclear risk of 49.9%, and high risk of 8.1% among all domains. The domains most susceptible to RoB were: (1) selection bias, with only 21.6% of studies reporting methods to generate random sequences, and 6.8% reporting allocation concealment; (2) performance bias with only 1.1% of studies reporting random housing; and (3) detection bias with only 2.3% of studies reporting random outcome assessment. Attrition and reporting domains instead had low degrees of bias (73.8% and 77.3%, respectively), indicating that outcome data were reported clearly and completely.

### MSC efficacy on the sensorimotor outcome

**Fig. 3:**
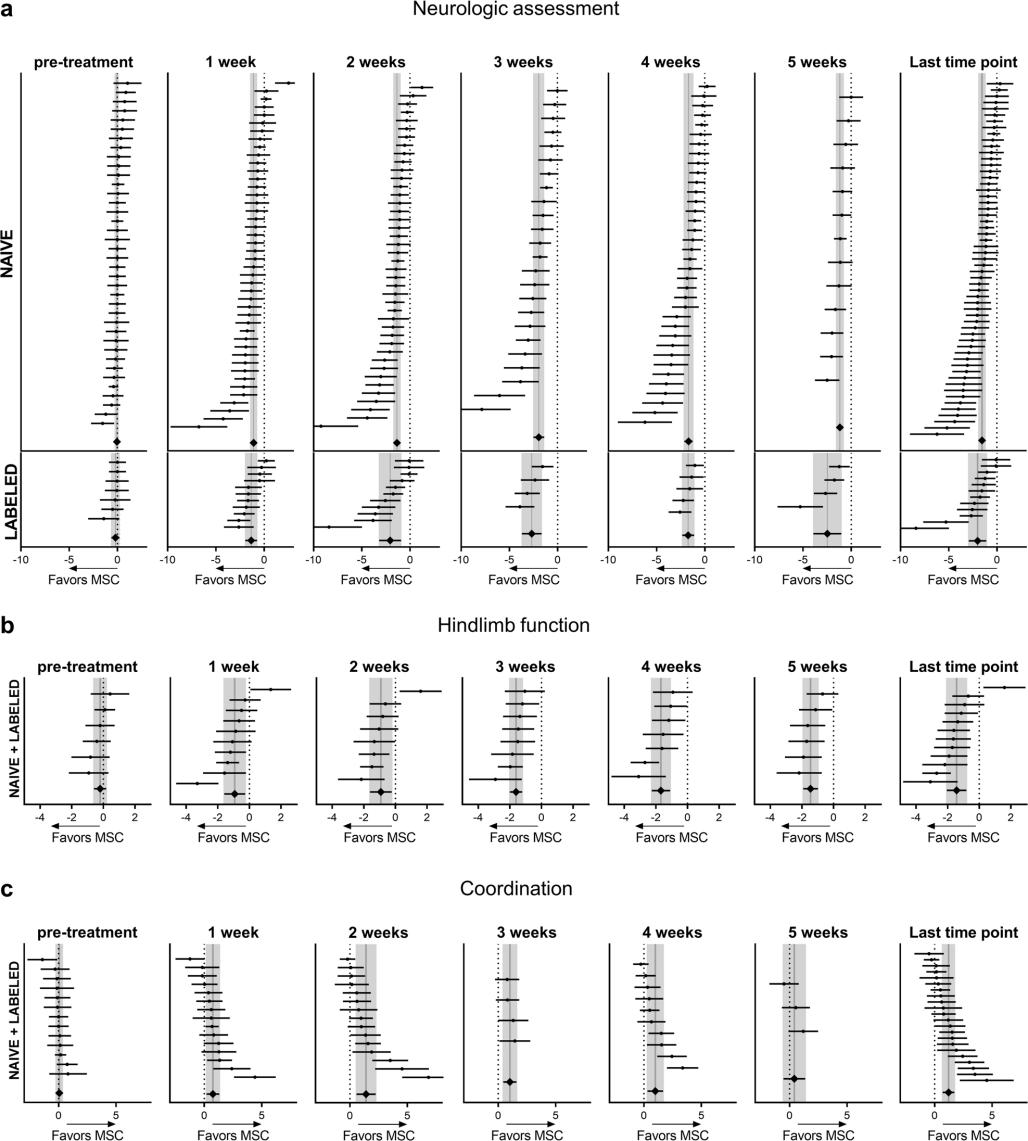
Sensorimotor outcome assessment up to 5 weeks post-treatment. Forest plots show mean effect size and 95% CI of naïve and labeled MSCs for neurologic assessments (**a**), hindlimb function (**b**), and coordination (**c**) up to 5 weeks post-treatment. The graph on the right shows the last time point (up to 5 weeks) for all studies. Dots represent the single studies, and diamonds indicate pooled data. Vertical gray bars represent the mean and 95% CI of the pooled estimated effect size.

First, we examined the effects of naïve and labeled MSCs on neurologic assessment over time, up to 5 weeks post-treatment (Fig. 3a, Supplementary Table 4). Naïve and labeled MSCs had similar effect sizes in favor of MSC treatment, starting 1 week post-treatment (naïve MSCs *p* < 0.001; labeled MSCs *p* < 0.001) and persisting up to 5 weeks (naïve MSCs *p* < 0.001; labeled MSCs *p* < 0.001). Since no differences were ever observed between naïve and labeled MSCs, data from these two groups were pooled for subsequent analysis. Heterogeneity among studies was substantial at 1 week (*I*^2^ 65%) but declined by 5 weeks post-treatment (*I*^2^ 45%).

For hindlimb function (Fig. 3b, Supplementary Table 4) there was a consistent over-time effect size in favor of MSCs, starting 1 week post-treatment (*p* = 0.006; *I*^2^ 73%) and persisting up to 5 weeks (*p* < 0.001; *I*^2^ 0%). Coordination assessment (Fig. 3c) showed a more variable effect size with the highest efficacy two weeks after treatment (*p* < 0.001; *I*^2^ 85%) but no efficacy at 5 weeks (*p* = 0.391; *I*^2^ 40%).

**Fig. 4:**
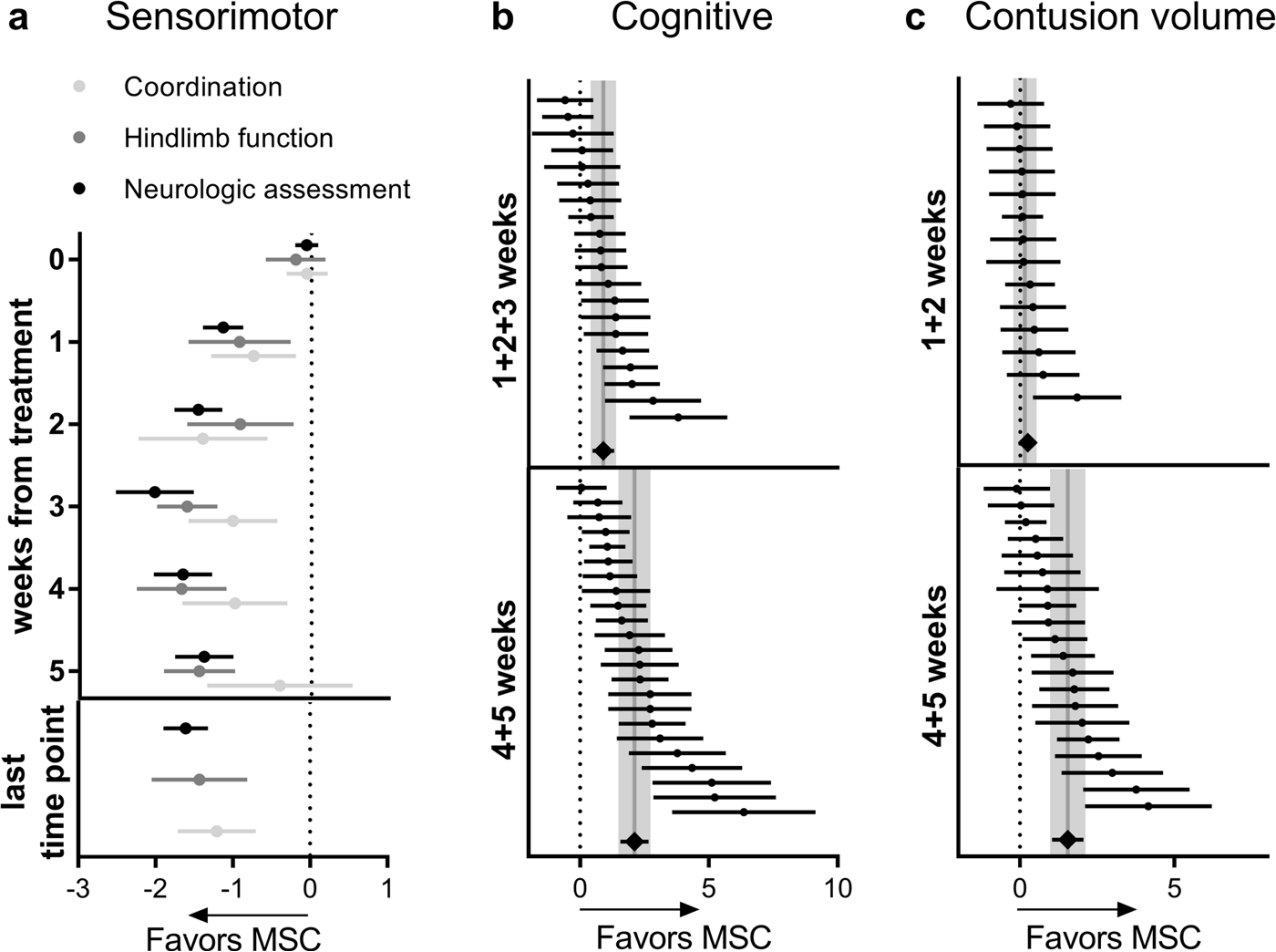
Summary of sensorimotor, cognitive, and anatomical outcomes. Forest plots show mean effect size and 95% CI of naïve+labeled MSCs for all sensorimotor tests (**a**), cognitive assessment (**b**), and contusion volume (**c**). Dots represent the single studies, and the diamonds represent pooled data. Vertical gray bars indicate the mean and 95% CI of the pooled estimated effect size.

Among sensorimotor outcomes, the neurological assessment showed a clear increase in effect size over time (Fig. 4a), peaking 3 weeks post-treatment (1 vs. 3 weeks *p* = 0.002). The other evaluations had similar temporal trends but with lower effect sizes (neurologic assessment vs. coordination *p* = 0.009 at 3 weeks).

### MSC efficacy on cognitive and anatomical outcomes

A favorable effect size of MSCs was found for cognitive function (Fig. 4b), both at 1–3 weeks (*p* < 0.001; *I*^2^ 59%) and 4–5 weeks post-treatment (*p* < 0.001; *I*^2^ 76%). Notably, the effect size at 4–5 weeks was larger than at earlier time points (*p* = 0.001). The efficacy of MSCs on anatomical damage only became evident 4–5 weeks post-treatment (Fig. 4c, *p* < 0.001, *I*^2^ 67%)

### Stratified meta-analysis

**Fig. 5:**
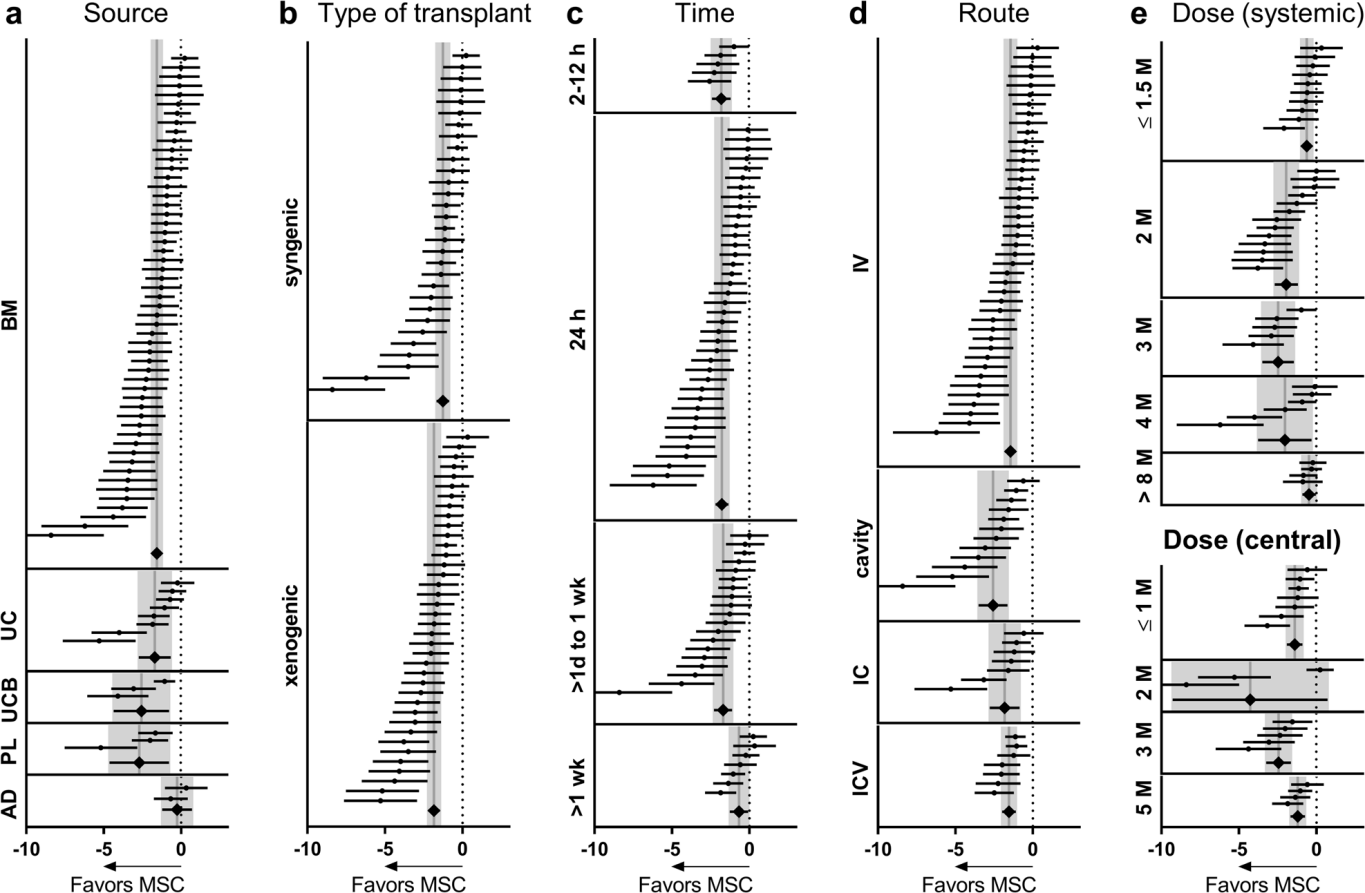
Subgroup meta-analysis on MSC-related variables. Forest plots of neurologic assessments at last time points of naïve+labeled MSCs stratified by source (**a**), type of transplant (**b**), time (**c**), and route of administration (**d**) and dose (**e**). Dots represent the single studies, and the diamonds represent pooled data. Vertical gray bars indicate the mean and 95% CI of the pooled estimated effect size.

Subgroup meta-analysis was done on the last time point of the neurologic assessment since this was when we had the largest number of comparisons. Analysis of the source of MSCs (Fig. 5a) showed significant effects of BM (*p* < 0.001), UC (*p* = 0.001), UCB (*p* = 0.006), and PL (*p* = 0.006) derived MSCs, while AD-derived MSCs had no effect (*p* = 0.600). Both syngeneic and xenogenic transplants induced a significant effect size in favor of treatment (*p* < 0.001), with no differences (Fig. 5b). For xenotransplants, we analyzed the effect of immunosuppressive treatment by comparing groups transplanting MSCs in the presence (*n* = 6) or without (*n* = 35) Cyclosporine A. There were no significant differences (*p* = 0.429, data not shown).

The therapeutic time window (Fig. 5c) showed a similar effect size in favor of MSCs when cells were transplanted at very acute times (1–12 h post-TBI, *p* < 0.001), at 24 h (*p* < 0.001) or sub-acutely (from 1 to 7 days post-TBI, *p* < 0.001). MSCs administered beyond 7 days was still effective (*p* = 0.026) although the effect size was smaller than with earlier administration (2h–7 days vs. >7 days *p* = 0.002).

Systemic (IV) and central delivery (into the cavity or intracranial (IC), or intracerebroventricular (ICV)) gave significant protection (Fig. 5d, *p* < 0.001) with greater effect size with transplant into the cavity over IV (*p* = 0.029).

Systemic infusion of any dose of MSCs resulted in significant effect size in favor of treatment (Fig. 5e). A dose-effect was found from 1.5 to 3 million (M) cells (*p* < 0.001), while the effect size was smaller at higher doses (>8 M vs. 3 M *p* < 0.001). A similar pattern was found for central delivery (1 M vs. 3 M *p* = 0.029), except for the 2 M dose, which showed wide variability.

MSCs were effective in both mice and rats (*p* < 0.001) with no significant differences (*p* = 0.355, Supplementary Fig. 1d). A significant sex effect was found (*p* < 0.001, Supplementary Fig. 1e), with greater effect size in males than females (*p* < 0.001). MSCs were effective in all TBI models (CCI, WD, and fluid percussion injury (FPI), Supplementary Fig. 1f). Last, no significant differences were found when comparing studies with low (<5) or high (≥5) quality scores (data not shown).

### Effects of MSC modifications to boost the efficacy

**Fig. 6:**
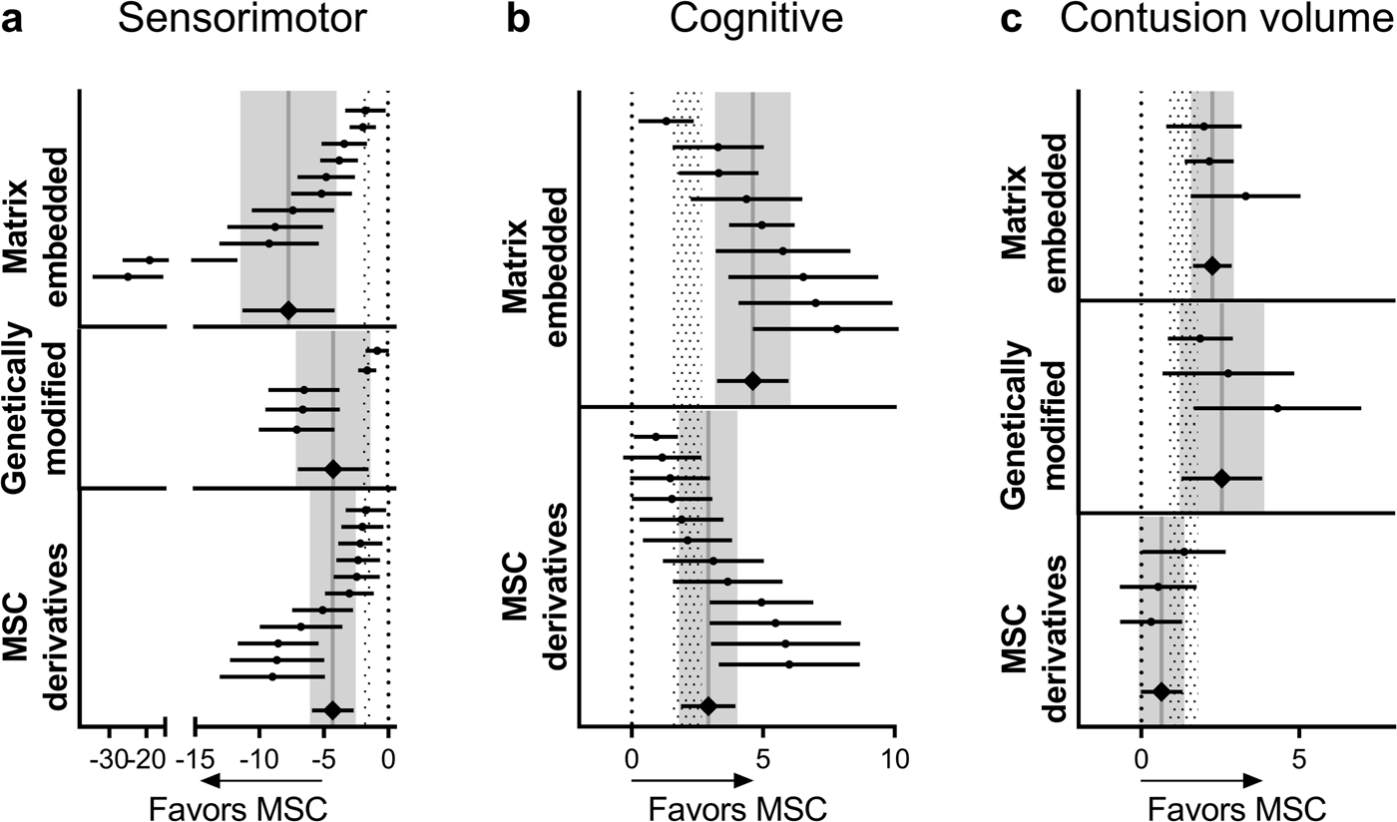
Effect of MSC modifications. Forest plots show mean effect size and 95% CI of genetically modified MSCs, matrix-embedded MSCs, and MSC derivatives for neurologic assessment (**a**), cognitive function at 4–5 weeks (**b**), and contusion volume at 4–5 weeks (**c**). Dots represent the single studies, and diamonds pooled data. Vertical pale gray bars indicate the mean and 95% CI of the pooled estimated effect size of the single modifications and vertical dotted bars represent 95% CI of the naïve + labeled MSC group.

The analysis of MSC modifications on neurologic assessment (Fig. 6a) detected significant effect sizes in favor of treatment compared to placebo for matrix-embedded (*p* < 0.001), genetically modified (*p* = 0.002), and MSC derivatives (*p* < 0.001). Matrix-embedded MSCs and MSC derivatives had greater effect size than unmodified MSCs (*p* < 0.001 and *p* = 0.001, respectively). Efficacy on cognitive function at 4–5 weeks (Fig. 6b) was confirmed for matrix-embedded MSCs (*p* < 0.001) and MSC derivatives (*p* < 0.001) with greater effects in matrix-embedded MSCs compared to unmodified MSCs (*p* < 0.01). Matrix-embedded MSCs and genetically modified MSCs also had a significant effect size on contusion volume at 4–5 weeks, in favor of treatment compared to placebo (*p* < 0.001, Fig. 6c). Matrix-embedded MSCs had greater effect size than unmodified MSCs (*p* < 0.05).

### Publication bias

**Fig. 7:**
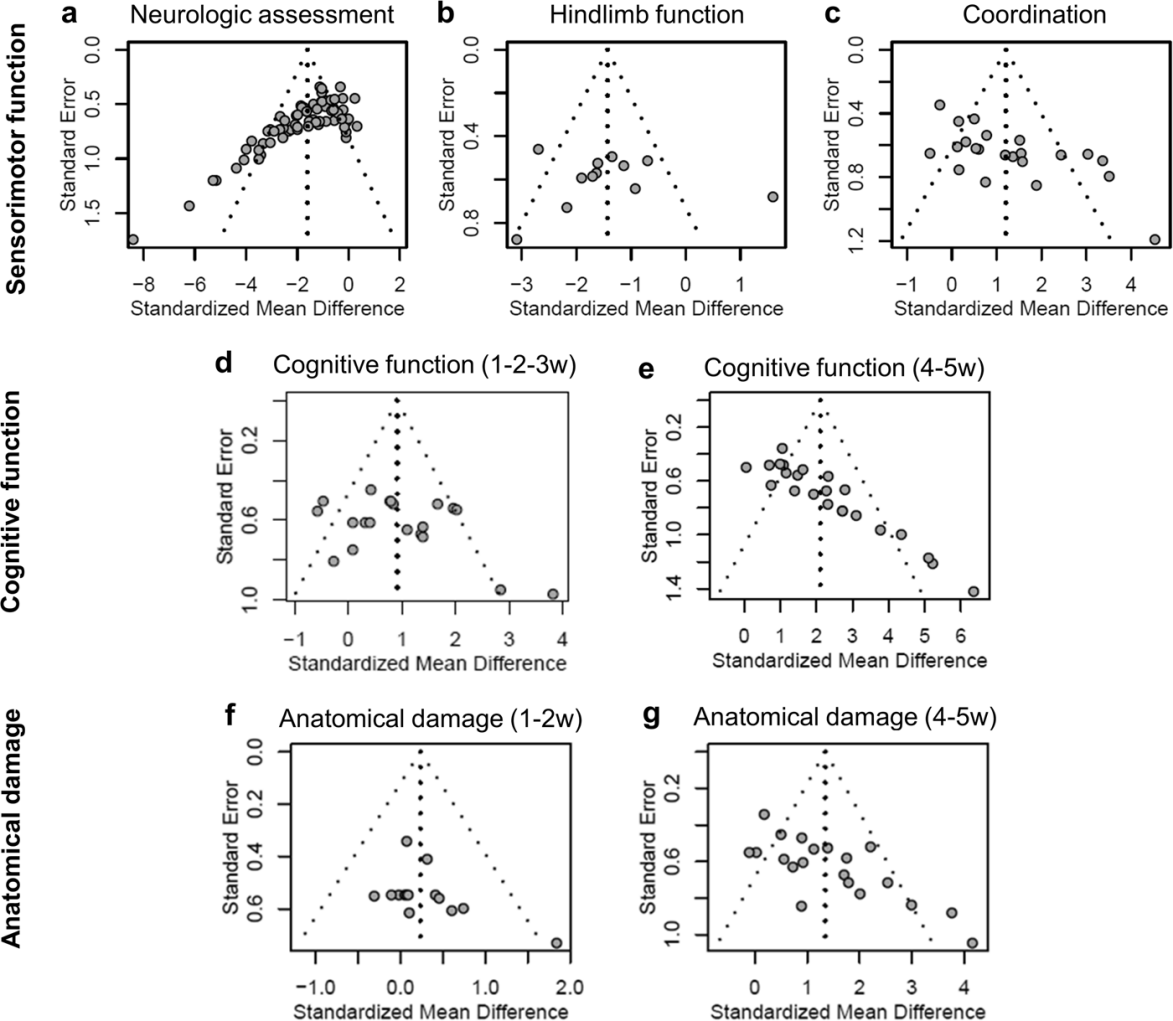
Begg’s funnel plots. Funnel plots representing publication bias for sensorimotor outcomes of naïve+labeled MSCs at the last time points: **a** neurological, **b** hindlimb function, **c** and coordination assessments. Publication bias for cognitive function of naïve+labeled MSCs assessed at early (**d**) or late (**e**) post-treatment time points. Publication bias for contusion volume at early (**f**) or late (**g**) post-treatment time points.

Publication bias of sensorimotor assessments was investigated at the last time point. Funnel plots showed asymmetry for neurologic assessment (Egger test *p* < 0.001, Fig. 7a) and coordination (*p* < 0.001, Fig. 7c) indicating publication bias, which was not present for hindlimb function (*p* = 0.755, Fig. 7b). Cognitive outcome presented publication bias at late (4–5 weeks, *p* < 0.001; Fig. 7e) but not early time points (1–3 weeks, *p* = 0.142; Fig. 7d). Similarly, contusion volume presented publication bias at late (4–5 weeks, *p* < 0.001; Fig. 7g) but not early time points (1–2 weeks, *p* = 0.125; Fig. 7f). All MSC modification groups presented publication bias for neurologic assessment (matrix-embedded *p* < 0.001, Supplementary Fig. 2a; genetically modified *p* = 0.020, Supplementary Fig. 2b; MSC derivatives *p* < 0.001, Supplementary Fig. 2c) and cognitive function (matrix-embedded *p* = 0.011, Supplementary Fig. 2d; MSC derivatives *p* < 0.001, Supplementary Fig. 2e) but not for contusion volume (matrix-embedded *p* = 0.478, Supplementary Fig. 2f; genetically modified *p* = 0.189, Supplementary Fig. 2g; MSC derivatives *p* = 0.346, Supplementary Fig. 2h).

## Discussion

**Fig. 8:**
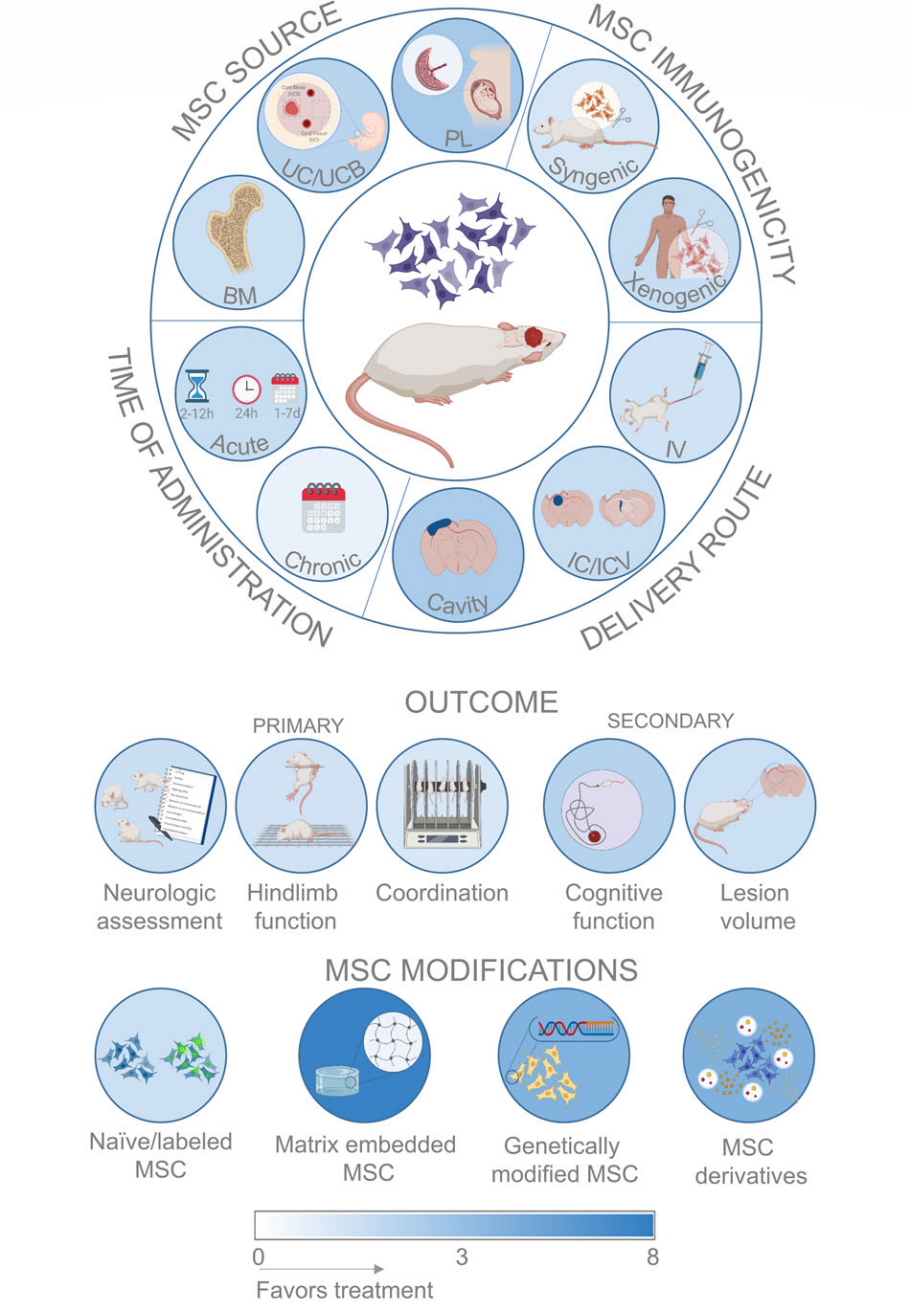
Graphical summary of the results, illustrating the main categories of variables in the meta-analysis. The top panel refers to the stratified meta-analysis on neurologic assessment using naïve + labeled MSCs. The background colors of the circles are indicative of the effect size (as absolute SMD) as in the color scale at the bottom. Image created with BioRender.com.

This meta-analysis investigated the effects of MSCs in preclinical models of TBI and found that they improved sensorimotor and cognitive functions as well as anatomical damage. Efficacy on sensorimotor outcomes was robust when analyzed in relation to MSC source and type of transplant, time of administration, delivery route, and doses (Fig. 8) as well as recipient species and multiple models of TBI. This supports the idea of clinical studies of MSCs for TBI patients. Our analysis also suggests that embedding MSCs into matrices or using MSC derivatives may boost efficacy (Fig. 8).

The current analysis found that MSCs improved sensorimotor function explored by three different tests, namely neurologic assessment, hindlimb function, and coordination. We did a longitudinal analysis to see whether some time is needed to observe significant effects after treatment. All sensorimotor evaluations indicated a significant effect size as early as 1 week post-treatment; however, at this time less than 50% of studies reported significant improvements. From 1 to 3 weeks after treatment, there was a significant increase in effect size for the neurologic assessment, with more than 80% of studies reporting significant effects at 3 weeks. At later times there were no further improvements or even a slight decrease. Therefore, when no effects are detected in the first 3 weeks after treatment, later improvements are unlikely. Spontaneous recovery after TBI might possibly reduce the difference between treated and untreated animals, contributing to the reduction of the effect size chronically.

Coordination, evaluated by the rotarod test, gave the lowest sensitivity and the greatest variability among sensorimotor tests, possibly due to the fast recovery of TBI animals to pre-injury values^102^. Our data are in line with a recent meta-analysis by Jackson et al.^103^ focusing on progenitor cells (not restricted to MSCs) in preclinical models of TBI, showing smaller absolute values of effect size achieved in the rotarod test (0.34) compared to Neurological Severity Score (1.36). Thus we do not recommend this as the first choice to test treatment efficacy.

MSC therapy improved cognitive function more when evaluated at late stages. Anatomical damage revealed a time-dependent relation, with the consistent absence of effects up to 2 weeks post-treatment but significant improvement after 1 month. These results support the notion that MSCs improve the injured microenvironment, mitigating progressive tissue loss. MSCs can reprogram the microenvironment, reducing detrimental pathways and stimulating endogenous neurorestorative ones, with the final effect of reducing the progression of the primary lesion^11,104,105^.

MSCs isolated from different sources, while having certain common features, may differ in several aspects, ranging from their phenotype to their functions, that could render the specific MSC line more suitable for a particular pathologic condition. Our meta-analysis indicated a similar improvement of sensorimotor function for all the sources except adipose tissue, with no differences between BM, UC, UCB, and PL. BM remains the most commonly used source, with UC as second, in line with clinical MSC studies^106^.

When MSCs were divided for their potential immunogenicity, the efficacy of syngeneic and xenogenic transplants was comparable. Among xenogenic transplants, three studies used concomitant immunosuppressive treatment, but with no further benefits in terms of efficacy. A direct comparison of MSCs’ effects in immunocompetent or immunosuppressed TBI animals confirmed there is no need for immunosuppressive treatment even after xenogenic MSC treatment^73^. These results have clinical implications since they support the use of allogeneic transplants in immunocompetent patients, allowing the use of bank-stored MSCs, immediately available for TBI patients, with no delay to therapy.

The effects of MSCs on sensorimotor function remained ample when administered within a few hours, and up to 1 week after TBI, whereas administration after 1 week was associated with smaller effect size, though still significant. The majority of studies testing chronic MSC treatment are by Bonilla and colleagues, who compared different delivery routes, and found efficacy when infused into the cavity^24,26,28^ but not intravenously^25^ or in the subarachnoid space^27^ two months post-TBI. Thus, a combination of time and route should give better information about chronic administration, and more studies are needed to validate these findings.

When analyzing acute treatment, our results indicate that all the routes offer protection on neurological function, but with different degrees of efficacy. MSCs delivered directly into the lesion cavity had greater effects than systemic delivery. Similar advantages were found in other meta-analyses investigating the effects of human cell therapy (not restricted to MSCs) in TBI models^107^ or MSCs in preclinical models of stroke^108^, in which transplantation into the lesion core was more effective than systemic administration. These data suggest that the presence of MSCs in the proximity of the lesion core may be of further benefit; however, in terms of clinical translatability, this approach is critical because of possible side effects related to the invasive procedure. Importantly, systemic delivery gave consistent therapeutic effects as well, and this is by far the most common and least invasive delivery route in clinical trials of both non-neurologic and neurologic conditions^106^, applicable for mild to severe TBI patients.

Systemic delivery gave an inverse U-shaped relation, with borderline significance with the lower dose (<1.5 M cells), a larger effect size with 2–4 M cells and less efficacy with higher doses. This relation was also observed for MSCs in stroke models^108^ and might be related to massive cell entrapment in capillaries, possibly causing organ dysfunction. Translation of MSC doses from experimental to clinical settings is not straightforward, but a recent analysis of MSCs used in the clinical setting indicated a minimal effective dose of 100–150 M cells, while doses below 70 M or above 200 M were less or not effective^106^, in line with the inverse U-shaped relation described here.

Last, MSCs showed efficacy across species and TBI models. Unexpectedly, we observed a significant sex difference, with male mice being more protected than females. This might be influenced by the fact that studies investigating chronic treatment, which already showed lower effect size, used only female rats, accounting for about one-third of the studies using female rodents (6/17). There is a clear bias toward male rodents in preclinical studies^109^, and sex differences in TBI pathophysiology and functional recovery are under-investigated^110^. Thus, additional studies are still needed to test MSCs’ efficacy in relation to sex.

In recent years different manipulations have been tried to boost MSCs’ therapeutic efficacy: genetic manipulation to overexpress or silence specific genes, in vitro preconditioning with different stimuli, or bioengineered scaffolds and matrices to improve MSC survival and engraftment. Recent evidence indicates that MSCs exert their therapeutic effects mainly through the release of bioactive factors in the media or shuttled into extracellular vesicles/exosomes, permitting tests of MSC-based cell-free strategies. The use of matrix-embedded MSCs was the most promising modification, with efficacy boosted compared to unmodified MSCs, verified for all the outcomes assessed. MSC derivatives showed boosted efficacy for neurologic assessment compared to unmodified MSCs. MSC-derived exosomes are now approaching the clinical setting, with pilot studies testing their safety and efficacy in neurological (Alzheimer disease and stroke) and non-neurological conditions (diabetes, cancer, and COVID-19, clinicaltrial.gov). In contrast, the use of matrices still awaits further steps for clinical approval, and the need for local transplantation is associated with limitations that call for a thorough evaluation of the risk-benefit balance.

Our study has the limitations inherent to systematic reviews^111^, including the fact that the quality of results is affected by the quality of single studies, and that we pooled data from studies with different characteristics, and this might explain part of the heterogeneity. Moreover, we detected clear publication bias in almost all the variables considered. Reluctance to publish negative results is the bias with the most impact, so the overall effect size could be overestimated. The quality assessment revealed the risk of selection bias and of underpowered studies, while RoB assessment indicated risks related to the absence of allocation concealment and randomization in the selection, housing, and outcome assessment of the animals. These data indicated the need for more rigorous experimental designs following the ARRIVE guidelines^112,113^, in order to improve translatability from bench to bedside. We selected widely used sensorimotor and cognitive functional tests to evaluate efficacy on neurological outcomes, and contusion volume as the measure of anatomical outcome. This meant that studies only relying on different outcome measures were excluded. However, this choice allowed us to limit the heterogeneity, run subgroup meta-analysis and draw inferences about the results from different studies and different laboratories.

Few clinical studies have assessed the safety, feasibility, and efficacy of MSC therapy for TBI patients so far^114–116^, and some are now ongoing^117^. The non-random, open-label interventional cohort study by Tian et al.^114^ (97 TBI patients) evaluated the safety and efficacy of 1 M autologous BM-MSCs transplanted by lumbar puncture in TBI patients one month or more after injury. The randomized, single-blind controlled trial by Wang et al.^115^ (20 controls and 20 MSC-treated TBI patients) examined the safety and efficacy of 10 M UC-MSCs transplanted by lumbar puncture, four times in 5–7 days at chronic stages (2–10 years after injury). Last, the double-blind, controlled phase 2 study by Kawabori et al.^116^ tested the effectiveness, safety, and tolerability of stereotactic intracranial implantation of allogenic modified BM-MSCs (SB623) in patients with stable chronic motor deficits secondary to TBI (1.4–28.4 years after injury). A total of 63 patients were randomized to control or SB623 treatment groups, at doses of 2.5, 5, or 10 M cells. All these studies reported safety and preliminary improvement of several neurological parameters.

In conclusion, our review documents a very large and favorable effect of MSCs on sensorimotor and cognitive functions and anatomical damage in preclinical models of TBI. The efficacy on sensorimotor outcomes remained robust across many variables related to MSC features (source, and type of transplant), treatment protocol (time and route of administration), and experimental model (host species and TBI model). From a clinical perspective, the analysis suggests an advantage of acute over chronic MSC treatment, with a smaller effect size when MSCs are given months after TBI. MSC transplantation at chronic stages is easier in terms of feasibility, but the mechanisms of action potentially induced by cell treatment are limited to the stimulation of neurodegenerative and plasticity pathways, while transplantation at acute stages could also target toxic pathways acutely triggered by the primary impact^118^.

The delivery route is pivotal. While we observed a greater effect size when MSCs were transplanted into the cavity, this route could add surgery-related risks, especially in the acute phase after TBI. Thus systemic MSC administration, which is minimally invasive, gives ample protection and has proved safe, may be preferred^106^.

## Methods

The study protocol was reviewed and registered on the PROSPERO database (registration number: CRD42020191403).

### Search strategy

Studies of MSCs in animal models of TBI were identified from electronic databases (PubMed and EMBASE, 4th June 2020). The queries and the research strings, applied with Boolean operators, are set out in Supplementary Table 5. The database enquiries were made by two independent subjects, and disagreements were solved after discussion with a third party.

### Inclusion criteria

Type of studies: we looked at comparative controlled studies assessing the efficacy of MSC-based interventions in preclinical models of TBI. Only articles in English were eligible. No restriction on the publication date was applied.

Preclinical models: Five TBI animal models were included, namely CCI, FPI, WD, closed head injury, and blast injury, for their reproducibility and clinical relevance. No restriction was applied on injury severity. Only studies using a single injury were included. The purpose of the study is to understand the efficacy of MSC therapy after TBI for future clinical studies on adults, so neonatal TBI models were excluded.

### Interventions

All studies using MSCs (allogeneic, syngeneic, or xenogeneic cells from any source tissue) or their derivatives as a therapeutic approach for TBI were eligible. To maintain clinically translatable validity, we included studies delivering MSCs at least 1 h after TBI induction; studies delivering MSCs before, during, or in the first hour after TBI were excluded. Any delivery route was accepted (IV, intra-arterial, ICV, IC, intraperitoneal, and intranasal). Multiple administration protocols were included.

We also intended to analyze whether specific modifications boosted MSC efficacy. Thus the following groups were identified: (1) Naïve: MSCs with standard culture protocols; (2) labeled: MSCs labeled with tracers or genetically modified to express fluorescent markers (e.g. GFP); (3) matrix-embedded: MSCs cultured and transplanted in 3D matrices (scaffolds or hydrogels of any material); (4) genetically modified: MSCs with genetic manipulations aimed at boosting their efficacy (not involving switching off RNA expression in order to confirm the involvement of that specific factor in the protection); (5) preconditioned: MSCs exposed to pre-conditioning stimuli before transplant (in vitro hypoxia, inflammatory cytokine or drug exposure, coculturing with brain homogenate or conditioned media from other cells); (5) MSC derivatives: studies using MSC-derived products (exosomes, extracellular vesicles, or secretome).

We excluded studies using MSCs differentiated into a specific linage or co-administered with other drugs or treatments.

### Comparators

The control intervention consisted of a placebo (saline, culture medium, PBS, or other vehicles).

### Data extraction

The following items were collected independently by two investigators: reference details (publication year and first author’s name); recipient animals’ characteristics (species, strain, sex, age, and weight); TBI model; type of anesthetic; analgesia, immunosuppression, and antibiotic; MSC characteristics (donor species and tissue source); intervention protocol (time of administration from injury, dose, route, number of doses); time and measures of outcome assessments. When a single publication reported more than one experimental group eligible for the meta-analysis, the data were collected and treated as independent experiments. When sensorimotor outcome tests were run longitudinally, all the time points were extracted. Data are reported as weeks from treatment onset, independently from the time between TBI and MSC delivery. Pre-treatment values (after injury but before MSC transplantation) were also extracted.

In case of missing data, we contacted the corresponding authors; if we received no reply, the study was excluded. If data were available only graphically, two independent investigators applied a digital ruler software (https://automeris.io/WebPlotDigitizer) to obtain numerical values.

### The methodological quality of the study and RoB

The study quality was assessed by two independent investigators, based on a checklist from the Collaborative Approach to Meta-Analysis and Review of Animal Data from Experimental Studies (CAMARADES)^20^. The checklist was slightly modified and defined as follows: (1) peer-reviewed, (2) temperature control during surgery; (3) treatment randomization; (4) blinded assessment of outcomes; (5) long-term assessments (≥28 days); (6) allocation concealment; (7) sample size calculation; (8) statement of welfare regulations for animal experimentation; and (9) statement on conflict of interest. One point was assigned for each quality criterion in the study.

To assess the RoB for each work, we used SYRCLE’s RoB checklist^119^.

### Outcomes

We aggregated tests assessing sensorimotor outcome as follows: (1) *neurologic assessment*: neurological severity score (NSS), modified NSS (mNSS), neuroscore; (2) *hindlimb function*: foot faults, beam walk, grid walk, and limb placing; (3) *coordination*: rotarod. For repeated measurements, all the time points were extracted separately. The earliest time point eligible was 1 week after treatment. For neurologic assessment and hindlimb function, the lower the score, the better the outcome; for coordination, the higher the score, the better the outcome. For studies using an inverse scale, the standardized mean difference (SMD) calculated was multiplied by −1.

Cognitive outcome was assessed by one of the following tests: Morris water maze (MWM), radial arm maze (RAM), radial arm water maze (RAWM), passive avoidance (PA), or novel object recognition (NOR). For MWM, RAM, and RAWM we extracted the data from the last day of the learning phase; for PA and NOR we extracted the data of the probe day.

The anatomical outcome was defined as the contusion volume evaluated by either histology or MRI.

Cognitive function evaluations and contusion volume data were pooled as sub-acute time points (1–3 weeks post-transplant) or later time points (4–5 weeks post-transplant). For both outcomes, the higher the score, the better the outcome. For studies using an inverse scale, the SMD calculated was multiplied by −1.

Only studies assessing at least one of these outcomes were analyzed.

### Data analysis

Only studies reporting the outcomes as mean and standard deviation (SD) or standard error were considered eligible for the meta-analysis. The overall effect of MSCs on outcomes was determined by calculating the SMD with 95% confidence intervals, using random-effect models^120^. SMD is equal to the difference in the mean outcome between treated and control groups divided by the standard deviation of outcomes among participants, reported in SD units. For sensorimotor outcome, we evaluated the effects of naïve and labeled MSCs over time and at the last available time, up to 5 weeks after treatment. Stratified meta-analysis and the effects of MSC modifications were checked at the last time point, up to 5 weeks. For modification groups, when there were fewer than three comparisons, no analysis was done. For this reason, the in vitro preconditioned MSC group is never present.

Studies reporting significant effect size at pre-treatment times were excluded from the meta-analysis. In studies with one control group compared to more than one experimental group, we adjusted the total numbers of control animals by dividing the number of animals in the control group by the number of treatment groups.

A few studies^26–28^ expressed efficacy as percentage changes from pre-treatment. In these cases, we computed the outcomes using as the starting point the mean of the absolute pre-treatment scores reported by the same authors in other publications^24,25^. We opted for a conservative approach and attributed to the means the highest SD provided in those publications.

Stratified analyses were also conducted to explore the influence of potential factors on the estimated effects of naïve and labeled MSCs on neurologic assessments, at the last time point. We stratified by source, type of transplant (syngeneic or xenogenic), time of administration, route, dose, rodent species, sex, TBI model, and study quality. Analysis of a possible dose effect was restricted to rats since they were used in most of the studies. Doses for systemic and central administration (as pooled data of cavity, IC, and ICV) were analyzed separately since they are not directly comparable.

Between-study heterogeneity was assessed using Cochran’s *Q* statistic, with a significant *Q* statistic (*p* < 0.05) indicating heterogeneity among studies, and using the *I*^2^ metric, with higher values denoting a greater degree of heterogeneity (0–40%: little heterogeneity; 30–60%: moderate heterogeneity; 50–90%: substantial heterogeneity; 75–100%: considerable heterogeneity)^120^.

Publication bias was investigated by visual inspection of funnel plots^121^ and using Egger’s test^122^.

All statistical analyses were done with the R-software version 3.4.1.

### Reporting summary

Further information on research design is available in the Nature Research Reporting Summary linked to this article.

## Supplementary information

Supplementary Information

Reporting Summary

## Data Availability

The full dataset used for the analysis is available at 10.5281/zenodo.5512613.

## Code availability

Custom codes are available from the corresponding author upon reasonable request.
