# Supplementary material for: Systematic review and meta-analysis of preclinical studies testing mesenchymal stromal cells for traumatic brain injury
Source: NPJ Regen Med. 2021 Oct 29;6:71. doi: 10.1038/s41536-021-00182-8 (PMC8556393; doi:10.1038/s41536-021-00182-8)
Supplement: Supplementary file 1 — Supplementary Information [file 41536_2021_182_MOESM1_ESM.pdf]

Table 1. Characteristics of the studies

reference	country	species	strain	sex	TBI model	anesthesia	immuno-suppression	antibiotics	analgesia	type of graft	MSC origin/ species	MSC origin/ organ	dose	route	time	manipulation	sensori-motor	cognitive	volume	total number of animals
Anbari et al 2014 [21]	Iran	rat	Wistar	M	WD	ketamine + cylazine	no	no	no	syngeneic	rat	BM	3M	IV	24 h	labeled	NSS	/	/	16
Bakhtiary et al 2010 [22]	Iran	rat	Wistar	M	CCI	chloral hydrate	no	no	no	allogeneic	rat SD	BM	2M	IV	24 h	labeled	mNSS	/	/	20
Bakhtiary et al 2011[23]	Iran	rat	Wistar	M	CCI	?	no	no	no	xenogenic	human	UC	2M	IV	24 h	labeled	mNSS	/	/	20
Bonilla et al 2009 [24]	Spain	rat	Wistar	F	WD	sevofluorane	no	no	morphine+ meloxicam	syngeneic	rat	BM	5M	cavity	60 d	naïve	mNSS rotarod	/	/	20
Bonilla et al 2012 [25]	Spain	rat	Wistar	F	WD	sevofluorane	no	no	morphine+ meloxicam	syngeneic	rat	BM	15M	IV	2 m	naïve	mNSS	/	HC	20
Bonilla et al 2012 [26]	Spain	rat	Wistar	F	WD	sevofluorane	no	no	morphine+ meloxicam	syngeneic	rat	BM	5M	cavity	2 m	naïve	mNSS	/	/	44
Bonilla et al 2014 [27]	Spain	rat	Wistar	F	WD	sevofluorane	no	no	morphine+ meloxicam	syngeneic	rat	BM	2M	SA	60 d	naïve	mNSS rotarod	/	HC	20
Bonilla et al 2018 [28]	Spain	rat	Wistar	F	WD	morphine + meloxicam	no	no	no	syngeneic	rat	BM	5M 1M	cavity	60 d	naïve matrix	mNSS rotarod	/	/	22
Chang et al 2013 [29]	Taiwan	rat	SD	M	FPI	pentobarbital	no	no	no	xenogenic	human	BM	100ug/ kg	IV	every 12 h	Pre-con CM	/	PA	HC	18
Chen et al 2014 [30]	China	rat	SD	M	WD	?	no	no	no	syngeneic	rat	BM	1M	IC	48 h	labelled	NSS	/	/	20
Cheng et al 2015 [31]	China	rat	SD	M	WD	chloral hydrate	no	no	no	xenogenic	human	PL	1mm3	cavity	24 h	naïve	mNSS	MWM	HC	12
Danilina et al 2017 [32]	Russia	rat	random-bred	?	WD	chloral hydrate	no	no	no	syngeneic	rat	BM	3M /kg	IV	24 h	naïve pre-cond	limb placing	/	MRI	44
Darkazalli et al 2016 [33]	USA	rat	SD	?	CCI	isoflurane	no	no	no	xenogenic	human	BM	1M	IV	6 h	naïve	/	NOR	MRI	16
Guan et al 2019 [34]	China	mouse	C57Bl/6	M	WD	?	no	no	no	xenogenic	human	UC	1M	IV	6+30+54 h	naïve	mNSS	MWM	HC	20
Guan et al 2013 [35]	China	rat	SD	M	CCI	chloral hydrate	no	no	Yes (not specified)	xenogenic	human	BM	3M	cavity	7 d	labeled matrix	mNSS	MWM	/	12
Han et al 2013 [36]	South Korea	rat	SD	M	FPI	xetamine + xylazine	no	no	no	xenogenic	human	BM	1M	IC	24 h 7d	naïve	NSS rotarod	/	/	27
Hong et al 2011 [37]	China	rat	SD	F	WD	isoflurane	no	Peni cillin	no	xenogenic	human	UC	0.1M	IC	7d	matrix	/	MWM	HC	40
Harting et al 2009 [38]	USA	rat	SD	?	CCI	Isoflurane	no	no	no	syngeneic	rat	BM	2M 4M	IV	24 h	labeled	NSS rotarod	MWM	/	15

reference	country	species	strain	sex	TBI model	anesthesia	immuno-suppression	antibiotics	analgesia	type of graft	MSC origin/ species	MSC origin/ organ	dose	route	time	manipulation	sensori-motor	cognitive	volume	total number of animals
Hu et al 2020 [39]	China	mouse	C57Bl/6	M	CCI	chrloral hydrate	no	no	no	xenogenic	human	UC	1M	IV	3 d	naïve	mNSS FF	MWM	HC	20
Hu et al 2019 [40]	China	rat	SD	M	WD	chloral hydrate	no	Peni cillin	no	syngeneic	rat	BM	0.1M	IC	24 h	labeled	mNSS	/	HC	12
Hu et al 2018 [41]	China	rat	SD	M	WD	chloral hydrate	no	no	no	syngeneic	rat	BM	1M	ICV	12 h	naïve	mNSS	/	/	12
Jiang et al 2011 [42]	USA	rat	Wistar	M	CCI	halothane	no	no	no	syngeneic	rat	BM	3M	cavity	7 d	matrix	mNSS	MWM	/	17
Kappy et al 2018 [43]	USA	rat	SD	M	CHI	ketamine+ xylaxine	no	no	no	xenogenic	human	AD	1M	IV	3 h	naïve	rotarod	/	/	30
Kim et al 2016 [44]	USA	mouse	C57BL/6J	M	CCI	sevofluorane	no	no	no	xenogenic	human	BM	30ug	IV	1 h	EV	/	MWM	/	25
Kim et al 2018 [45]	South Korea	mouse	C57BL/6J	M	CCI	?	no	no	no	xenogenic	human	PL	0.2M	IV	4+24 h 24h	naïve	rotarod	/	/	16
Kim et al 2010 [46]	South Korea	rat	SD	M	CCI	isoflurane	no	no	no	xenogenic	human	BM	2M	IV	24 h	naïve	mNSS rotarod	/	/	64
Kota et al 2016 [47]	USA	rat	SD	M	CCI	isoflurane	no	no	no	xenogenic	human	BM	1M/kg	IV	72 h	naïve	/	MWM	/	12
Lam et al 2016 [48]	China	rat	SD	M	CCI	ketamine	no	no	no	syngeneic	rat	AD	1.5M	cavity	1 h	labeled	rotarod	MWM	/	20
Li et al 2011 [49]	USA	rat	Wistar	M	CCI	chloral hydrate	no	no	burprenor-phine	xenogenic	human	BM	3M	IV	5 d	labeled	mNSS	MWM	MRI	18
Li et al 2012 [50]	USA	rat	Wistar	M	CCI	chloral hydrate	no	no	burprenor-phine	xenogenic	human	BM	3M	IV	6 h	naïve	mNSS	MWM	MRI	18
Li et al 2017 [51]	USA	rat	Wistar	M	CCI	chloral hydrate	no	no	burprenor-phine	xenogenic	human	BM	3M	IV	6 h 7 d	naïve	mNSS	/	MRI	20
Li et al 2017 [52]	USA	rat	Wistar	M	CCI	chloral hydrate	no	no	burprenor-phine	xenogenic	human	BM	3M	IV	6 h 7 d	naïve	/	MWM	MRI	30
Lin et al 2019 [9]	Taiwan	rat	SD	M	FPI	ketamine+ atropine+ xylazine	no	no	burprenor-phine	xenogenic	human	UC	1M 4M	IV	24 h	naïve	mNSS	/	HC	20
Liu et al 2014 [53]	China	rat	SD	M	WD	chloral hydrate	no	no	no	syngeneic	rat	BM	?	ICV	24 h	labeled	BW	/	/	24
Lu et al 2001 [10]	USA	rat	Wistar	M	CCI	chloral hydrate	no	no	no	syngeneic	rat	BM	2M	IV	24 h	labeled	NSS rotarod	/	/	12
Lu et al 2002 [54]	USA	rat	Wistar	?	CCI	chloral hydrate	no	no	no	xenogenic	human	UCB	2M	IV	24 h	naïve	NSS rotarod	/	/	16
Lu et al 2007 [55]	USA	rat	Wistar	M	CCI	chloral hydrate	no	no	no	xenogenic	human	BM	3M	cavity	4 d	naïve matrix	mNSS	MWM	HC	12

reference	country	species	strain	sex	TBI model	anesthesia	immuno-suppression	antibiotics	analgesia	type of graft	MSC origin/ species	MSC origin/ organ	dose	route	time	manipulation	sensori-motor	cognitive	volume	total number of animals
Lu et al 2003 [56]	USA	rat	Wistar	M	CCI	chloral hydrate	no	no	no	syngeneic	rat	BM	1M 2M 4M	IV	24 h	naïve	mNSS	/	/	18
Ma et al 2015 [57]	China	rat	SD	M	CCI	chloral hydrate	CsA	no	no	xenogenic	human	UC	10M	IV	24 h	labeled	mNSS	/	/	?
Mahmood et al 2001 [58]	USA	rat	Wistar	F	CCI	chloral hydrate	no	no	no	syngeneic	rat	BM	2M	IV	24 h	naïve	NSS rotarod	/	/	8
Mahmood et al 2002 [59]	USA	rat	Wistar	M	CCI	chloral hydrate	no	no	no	syngeneic	rat	BM	1M	IC	24 h	labeled pre-cond	rotarod	/	/	12
Mahmood et al 2003 [60]	USA	rat	Wistar	M	CCI	chloral hydrate	no	no	no	xenogenic	human	BM	1M 2M	IV	24 h	naïve	mNSS, rotarod	/	/	18
Mahmood et al 2004 [61]	USA	rat	Wistar	M F	CCI	chloral hydrate	no	no	no	syngeneic	rat	BM	1M 2M	IC IV	24 h	naïve	mNSS, rotarod	/	/	26
Mahmood et al 2005 [62]	USA	rat	Wistar	M	CCI	chloral hydrate	no	no	no	xenogenic	human	BM	2M 4M 8M	IV	24 h	naïve	NSS	/	/	40
Mahmood et al 2006 [63]	USA	rat	Wistar	F	CCI	chloral hydrate	no	no	no	syngeneic	rat	BM	2M 4M 8M	IV	7 d	naïve	NSS	/	/	20
Mahmood et al 2007 [64]	USA	rat	Wistar	F	CCI	chloral hydrate	no	no	no	syngeneic	rat	BM	1M	IV	24 h	naïve	mNSS	MWM	/	16
Mahmood et al 2008 [65]	USA	rat	Wistar	F	CCI	chloral hydrate	no	no	no	syngeneic	rat	BM	1M 2M	IV	7 d	naïve	mNSS	/	/	16
Mahmood et al 2013 [66]	USA	rat	Wistar	M	CCI	chloral hydrate	no	no	no	xenogenic	human	BM	3M	cavity	7 d	naïve matrix	mNSS FF	MWM	/	16
Maruichi et al 2009 [67]	Japan	rat	SD	M	CHI	isoflurane	CsA	no	no	xenogenic	mice	BM	0.4M	IC	10 d	labeled genetic	/	MWM	/	19
Mastro-Martinez et al 2015 [68]	Spain	rat	SD	M	WD	ketamine, diazepam, atropina	no	no	tramadol	syngeneic	rat	AD	0.2M	IC	24 h	naïve	rotarod	/	HC	20
Mishra et al 2017 [69]	India	mouse	Balb/c	M	WD	ketamine-xylazine	no	no	no	syngeneic	mice	BM	1.25M	IV	24 h	naïve	/	NOR	/	20
Nichols et al 2013 [70]	USA	rat	SD	M	FPI	isoflurane	no	no	Bupiva-caine	xenogenic	human	periph. blood	0.5M	ICV	24 h	labeled	/	MWM	/	16

reference	country	species	strain	sex	TBI model	anesthesia	immuno-suppression	antibiotics	analgesia	type of graft	MSC origin/ species	MSC origin/ organ	dose	route	time	manipulation	sensori-motor	cognitive	volume	total number of animals
Patel et al 2018 [71]	USA	rat	Fisher	M	CCI	isoflurane	no	No	Ketoprofen	xenogenic	human	AD	100ug	IV	3 h	EV	/	/	HC	38
Peruzzaro et al 2019 [72]	USA	rat	SD	M	CCI	isoflurane	no	no	no	syngeneic	rat	BM	0.8M	IC	36 h	labeled genetic	rotarod BW	MWM	/	29
Pischiutta et al 2014 [73]	Italy	mouse	C57BL/6	M	CCI	pentobarbital	no CsA	no	no	xenogenic	human	BM	0.15M	ICV	24 h	naïve	NS BW	MWM	HC	24
Pischiutta et al 2016 [74]	Italy	mouse	C57Bl/6	M	CCI	pentobarbital	no	no	no	xenogenic	human	PL	0.15M 1M	ICV IV	24 h	naïve	NS, BW	/	/	24
Qu et al 2008 [75]	USA	mouse	C57BL/6J	F	CCI	chloral hydrate	no	no	no	syngeneic	mouse	BM	0.3M	IV	24 h	naïve	FF	MWM	HC	12
Qu et al 2009 [76]	USA	mouse	C57BL/6J	M	CCI	chloral hydrate	no	no	no	xenogenic	human	BM	0.3M	Cavity IV	7 d	naïve matrix	/	MWM	HC	40
Qu et al 2011 [77]	USA	rat	Wistar	M	CCI	chloral hydrate	no	no	no	xenogenic	human	BM	3M	cavity	7 d	naïve matrix	mNSS	MWM	HC	16
Ruppert et al 2020 [78]	USA	rat	SD	M	CCI	isoflurane	no	no	no	xenogenic	human	AD	3M /kg	IV	3 d 14 d	naïve	NS	MWM	/	23
Shahror et al 2020 [79]	Taiwan	mouse	C57BL	M	CCI	zolasepam xilazine	no	no	no	syngeneic	mouse	BM	0.015M	ICV	24 h	genetic	/	MWM NOR	/	24
Shi et al 2018 [80]	China	mouse	Balb/c	M	CCI	isoflurane	no	no	Burprenor-phine	syngeneic	mouse	BM	1M	IV	6 h	genetic	rotarod	/	/	16
Shi et al 2018 [81]	China	mouse	Balb/c	M	CCI	isoflurane	no	no	Burprenor-phine	syngeneic	mouse	BM	2M	IV	6 h	naïve genetic	mNSS	/	HC	?
Shin et al 2016 [82]	Korea	rat	SD	M	CCI	zolazepam tiletamine	no	no	no	syngeneic	rat	BM	0.1M	IC	7 d	naïve	/	PA RAM	/	20
Silachev et al 2015 [83]	Russia	rat	random bread	?	WD	chloral hydrate	no	no	no	syngeneic	rat	BM	1.5M	IV IA	24 h	naïve	NSS	/	MRI	28
Tajiri et al 2014 [84]	USA	rat	Fisher	M	CCI	isoflurane	no	No	Ketoprofen	xenogenic	human	AD	4M	IV	3 h	Naïve CM	/	RAWM	HC	51
Turtzo et al 2015 [85]	USA	rat	Wistar	F	CCI	isofluorane	no	no	Acetamino-phen	syngeneic	rat	BM	5M x3	IV	3+5+7 d	naïve	NSS rotarod	/	MRI	34
Wang et al 2013 [86]	USA	rat	Wistar	M	CCI	?	no	no	no	xenogenic	human	BM	3M	IV	6 d	naïve	mNSS	MWM	/	15
Wang et al 2013 [87]	China	rat	SD	F	FPI	chloral hydrate	no	no	no	syngeneic	rat	BM	1M	ICV	24 h	naïve genetic	NSS	/	/	60
Wang et al 2015 [88]	USA	mouse	BALB/c	M	CCI	isofluorane	no	no	Burprenor-phine	allogeneic	FVB mice	BM	0.5M	IV	24 h	naïve genetic	rotarod	/	/	20-24

reference	country	species	strain	sex	TBI model	anesthesia	immuno-suppression	antibiotics	analgesia	type of graft	MSC origin/ species	MSC origin/ organ	dose	route	time	manipulation	sensori-motor	cognitive	volume	total number of animals
Wu et al 2019 [17]	China	mouse	?	M	CCI	chloral hydrate	no	no	no	xenogenic	rat	BM	0.3M	IC	24 h	labeled genetic	mNSS rotarod	/	HC	20
Xiong et al 2009 [89]	USA	rat	Wistar	M	CCI	chloral hydrate	no	no	no	xenogenic	human	BM	3M	cavity	7 d	naïve matrix	mNSS	MWM	/	12
Xu et al 2020 [90]	China	rat	SD	M	CCI	chloral hydrate	no	no	no	syngeneic	rat	BM	100ug	IV	24 h	EV	mNSS	MWM	/	12
Xu et al 2014 [91]	China	rat	SD	M	WD	chloral hydrate	no	no	no	syngeneic	rat	BM	1M	IC	48 h	labeled	NSS	/	/	20
Xu et al 2018 [16]	China	mouse	C57Bl/6	M	WD	chloral hydrate	no	no	no	xenogenic	human	UC	1M x3	IV	24+48+ 72 h	naïve genetic	mNSS	MWM	HC	30
Yan et al 2019 [14]	China	rat	Wistar	M	WD	chloral hydrate	no CsA	no	no	syngeneic	rat	BM	2M	cavity	72 h	labeled matrix	mNSS	MWM	/	20
Yan et al 2013 [92]	China	rat	SD	F	WD	isoflurane	no	penici llin	no	xenogenic	human	PL	0.1M	IC	4 d	matrix	mNSS	MWM	HC	20
Yao et al 2019 [15]	China	rat	SD	?	WD	?	no	Peni-cillin	no	xenogenic	mouse	BM	?	cavity	7 d	naïve matrix	mNSS	MWM	/	12
Yuan et al 2020 [18]	China	mouse	C57BL/6J	M	CCI	isoflurane	no	no	no	syngeneic	mouse	BM	2M	IV	24 h	naïve pre-cond	grid walk	MWM	HC	16
Yuan et al 2014 [93]	China	rat	SD	F	WD	chloral hydrate	CsA	Peni-cillin	no	xenogenic	human	UC	2M	IC	24 h	labeled genetic	NSS	/	/	16
Zanier et al 2011 [94]	Italy	mouse	C57BL/6J	M	CCI	pentobarbital	CsA	no	no	xenogenic	human	UCB	0.15M	ICV	24 h	naïve	NS BW	MWM	HC	36
Zanier et al 2014 [95]	Italy	mouse	C57BL/6J	M	CCI	pentobarbital	no	no	no	xenogenic	human	BM	0.15M	ICV	24 h	labeled	NS BW	/	/	16
Zhao et al 2012 [96]	China	rat	SD	M	WD	chloral hydrate	no	no	no	xenogenic	human	UCB	3M	IV	24 h	naïve	NSS	/	/	12
Zhang et al 2013 [97]	China	rat	SD	M	WD	pentobarbital	no	no	no	syngeneic	rat	BM	4M	IV	2 h	naïve	mNSS	/	/	12
Zhang et al 2015 [98]	USA	rat	Wistar	M	CCI	chloral hydrate	no	no	no	syngeneic	rat	BM	100ug	IV	24 h	EV	mNSS FF	MWM	HC	16
Zhang et al 2017 [99]	USA	rat	Wistar	M	CCI	chloral hydrate	no	no	no	xenogenic	human	BM	100ug	IV	24 h	EV	mNSS FF	MWM	HC	16
Zhang et al 2020 [100]	USA	rat	Wistar	M	CCI	ketamine-xylazine	no	no	no	xenogenic	human	BM	50ug 100ug 200ug	IV	24 h 4d 7d	EV	mNSS FF	MWM	/	16
Zhou et al 2016 [101]	China	rat	SD	M/F	FPI	urethane	no	no	no	xenogenic	human	UC	1M	IV	24 h	naïve	mNSS	MWM	/	20

Supplementary Figure 1

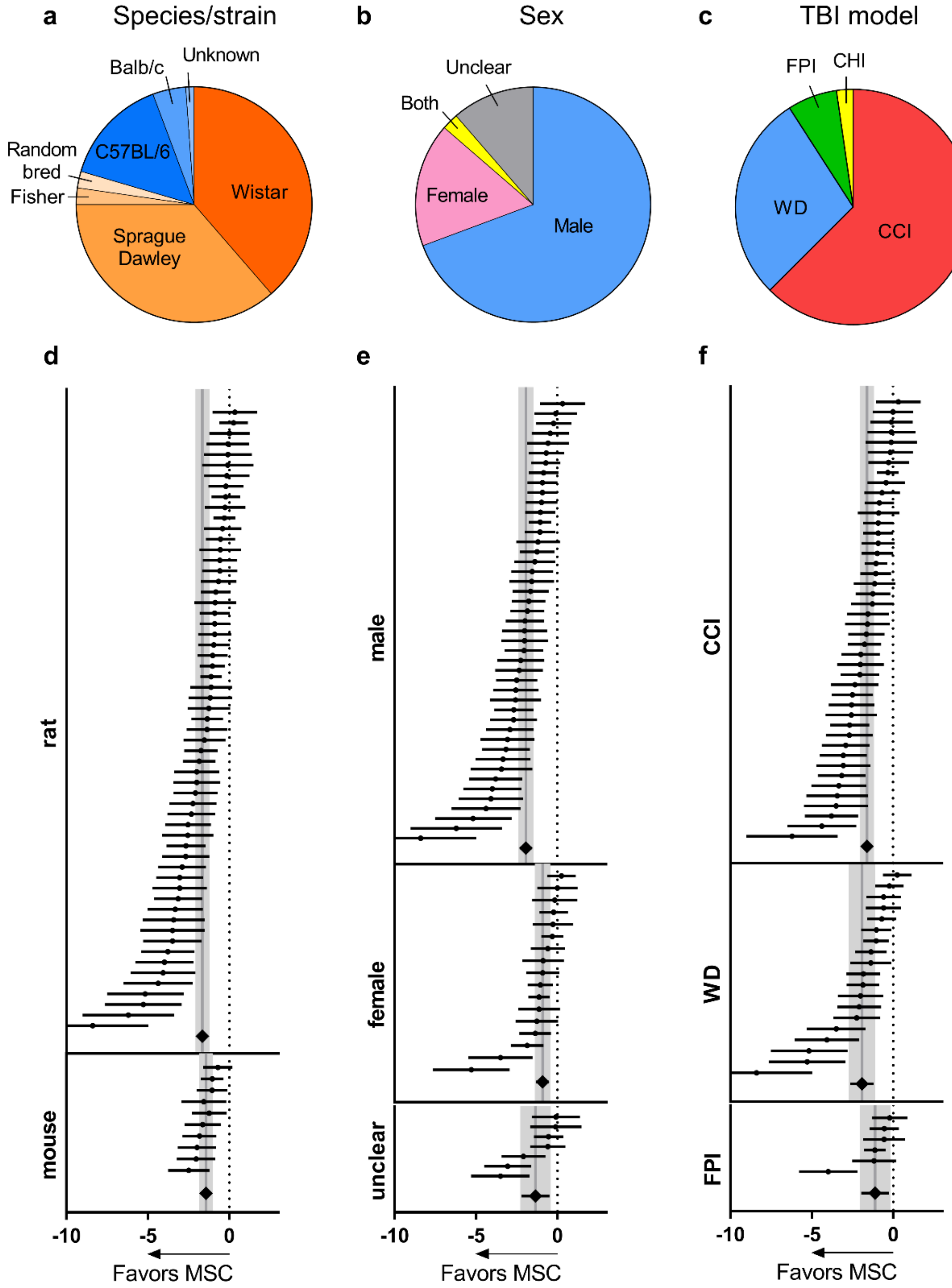

Supplementary Figure 1: Model-related characteristics and their subgroup meta-analysis. Pie charts of model-related features concerning species/strain (**a**), sex (**b**) and TBI model (**c**) of the 88 studies included in the qualitative synthesis. Forest plots of neurologic assessments of the last time point of naïve+labeled MSC stratified by species (**d**), sex (**e**) and TBI model (**f**). Dots represent the single studies, and the diamonds pooled data. Vertical gray bars represent the mean and 95% CI of the pooled estimated effect size.

Supplementary Figure 2

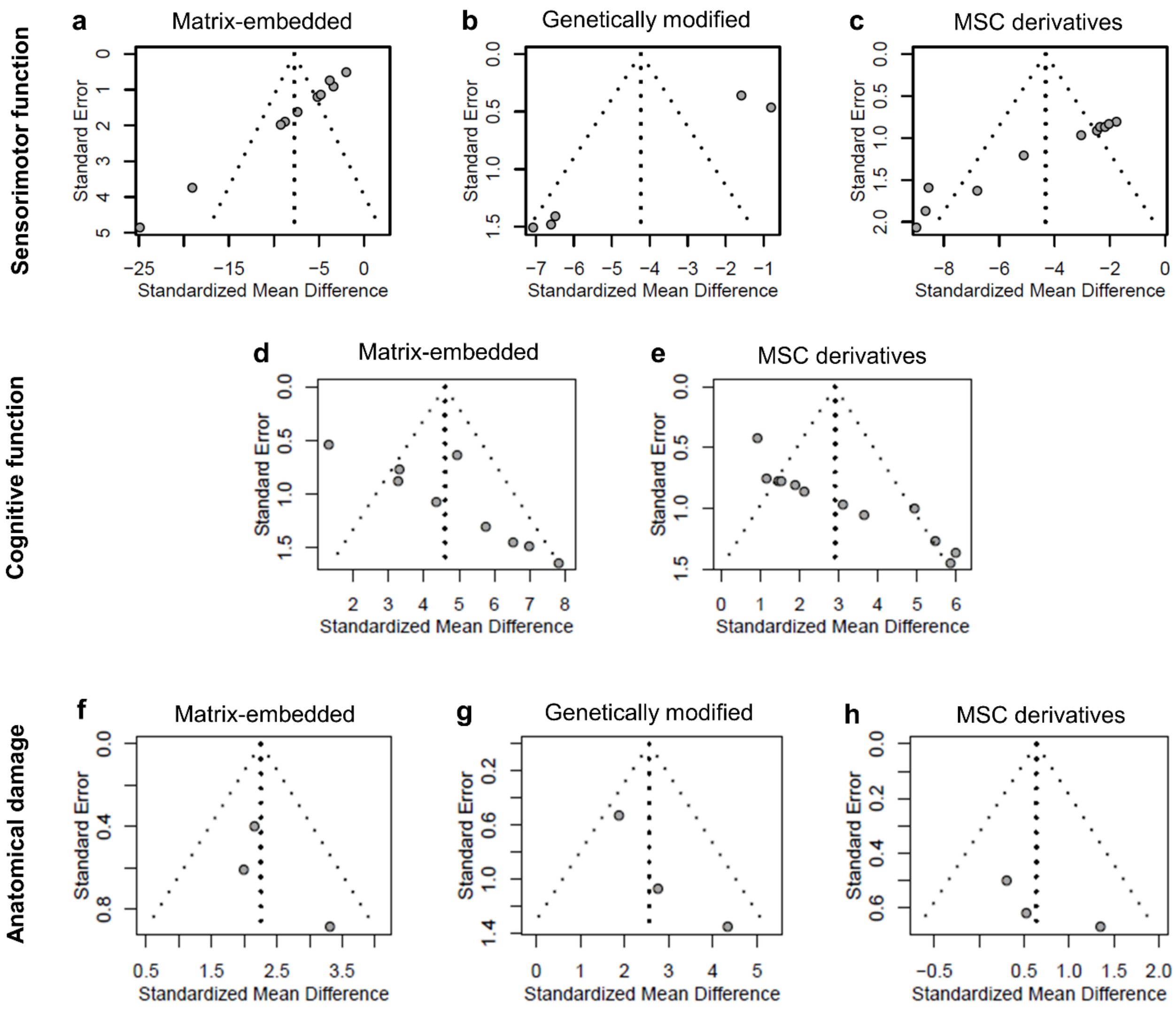

Supplementary Figure 2: Begg's funnel plots for MSC modifications. Funnel plots representing publication bias for neurological assessment at the last time points of matrix-embedded MSCs (**a**), genetically modified MSCs (**b**) and MSC derivatives (**c**). Publication bias for cognitive function at 4-5 weeks of matrix-embedded MSCs (**d**) and MSC derivatives (**e**). Publication bias for contusion volume at 4-5 weeks of matrix-embedded MSCs (**f**), genetically modified MSCs (**g**) and MSC derivatives (**h**).

Supplementary Table 2: Quality Score of studies

Author/year	peer rev	temperature control	treatment randomization	blinded outcome assasment	long-term assessment	allocation concealment	sample size calulation	welfare regulation	conflict of interest	SUM
Anbari F 2014	✓		✓					✓	✓	4
Bakhtiary M 2010	✓	✓	✓	✓	✓			✓		6
Bakhtiary M 2011	✓			✓	✓			✓		4
Bonilla C 2009	✓		✓		✓			✓	✓	5
Bonilla C 2012a	✓		✓		✓			✓	✓	5
Bonilla C 2012b	✓	✓	✓		✓			✓	✓	6
Bonilla C 2014	✓	✓	✓		✓			✓	✓	6
Bonilla C 2018	✓	✓	✓		✓			✓	✓	6
Chang CP 2013	✓	✓	✓	✓				✓		5
Chen L 2014	✓		✓		✓					3
Cheng T 2015	✓	✓	✓	✓	✓			✓	✓	7
Danilina TI 2017	✓	✓	✓					✓		4
Darkazalli A 2016	✓	✓						✓		3
Guan F 2019	✓	✓	✓	✓	✓			✓	✓	7
Guan J 2013	✓		✓	✓	✓			✓		5
Han EY 2013	✓		✓	✓	✓			✓	✓	6
Harting MT 2009	✓	✓		✓				✓	✓	5
Hong SQ 2011	✓	✓			✓			✓		4
Hu H 2020	✓		✓	✓				✓	✓	5
Hu J 2019	✓	✓	✓	✓	✓			✓	✓	7
Hu W 2018	✓		✓	✓				✓	✓	5
Jiang Q 2011	✓			✓	✓			✓		4
Kappy NS 2018	✓		✓					✓	✓	4
Kim C 2018	✓	✓	✓		✓			✓		5
Kim DK 2016	✓	✓			✓			✓	✓	5
Kim HJ 2010	✓	✓			✓			✓	✓	5
Kota DJ 2015	✓			✓	✓			✓	✓	5
Lam PK 2016	✓							✓	✓	3
Li L 2011	✓	✓	✓	✓	✓			✓	✓	7
Li L 2012	✓	✓	✓	✓	✓			✓	✓	7
Li L 2017	✓	✓	✓	✓				✓	✓	6
Li L 2017	✓	✓	✓		✓			✓	✓	6
Lin CH 2019	✓	✓	✓		✓			✓	✓	6
Liu Y 2014	✓				✓			✓		3
Lu D 2001	✓	✓	✓	✓						4
Lu D 2002	✓	✓		✓	✓			✓		5
Lu D 2007	✓	✓	✓	✓	✓			✓		6
Lu M 2003	✓						✓			2

Author/year	peer rev	temperature control	treatment randomization	blinded outcome assassment	long-term assessment	allocation concealment	sample size calulation	welfare regulation	conflict of interest	SUM
Ma J 2015	✓			✓					✓	3
Mahmood A 2001	✓	✓	✓	✓						4
Mahmood A 2002	✓	✓	✓	✓						4
Mahmood A 2003	✓	✓		✓	✓			✓		5
Mahmood A 2004b	✓	✓	✓	✓						4
Mahmood A 2005	✓	✓		✓	✓			✓		5
Mahmood A 2006	✓	✓		✓	✓			✓		5
Mahmood A 2007	✓	✓		✓	✓					4
Mahmood A 2008	✓	✓		✓	✓			✓		5
Mahmood A 2013	✓		✓	✓	✓			✓	✓	6
Maruichi K 2009	✓	✓			✓			✓		4
Mastro-Martinez I 2015	✓	✓	✓	✓				✓	✓	6
Mishra SK 2017	✓		✓	✓				✓	✓	5
Nichols JE 2013	✓		✓					✓	✓	4
Patel NA 2018	✓	✓	✓	✓				✓	✓	6
Peruzzaro ST 2019	✓	✓	✓					✓	✓	5
Pischiutta F 2014	✓	✓		✓	✓	✓		✓	✓	7
Pischiutta F 2016	✓	✓		✓	✓	✓		✓	✓	7
Qu C 2008	✓	✓	✓	✓	✓			✓		6
Qu C 2009	✓	✓	✓		✓			✓		5
Qu C 2011	✓	✓	✓	✓				✓		5
Ruppert KA 2020	✓				✓			✓		3
Sharor RA 2020	✓	✓			✓			✓	✓	5
Shi X 2018a	✓	✓						✓	✓	4
Shi X 2018b	✓	✓						✓	✓	4
Shin MS 2016	✓				✓			✓	✓	4
Silachev DN 2015	✓	✓	✓					✓		4
Tajiri 2014	✓	✓		✓				✓	✓	5
Turtzo LC 2015	✓		✓		✓			✓	✓	5
Wang S 2013	✓			✓	✓			✓	✓	5
Wang Z 2013	✓	✓		✓	✓					4
Wang Z 2015	✓	✓						✓	✓	4
Wu K 2019	✓		✓	✓	✓			✓	✓	6
Xiong Y 2009	✓	✓	✓	✓	✓			✓		6
Xu H 2020	✓				✓			✓	✓	4
Xu HS 2014	✓		✓		✓			✓	✓	5
Xu L 2019	✓		✓	✓	✓			✓	✓	6
Yan F 2019	✓		✓		✓			✓	✓	5
Yan ZJ 2013	✓	✓			✓			✓	✓	5
Yao M 2019	✓	✓	✓	✓	✓			✓	✓	7

Author/year	peer rev	temperature control	treatment randomization	blinded outcome assassment	long-term assessment	allocation concealment	sample size calulation	welfare regulation	conflict of interest	SUM
Yuan X 2020	✓		✓	✓	✓			✓	✓	**6**
Yuan Y 2014	✓			✓				✓	✓	**4**
Zanier ER 2011	✓	✓		✓	✓	✓		✓	✓	**7**
Zanier ER 2014	✓	✓	✓	✓	✓	✓		✓	✓	**8**
Zhang J 2012	✓		✓	✓	✓			✓	✓	**6**
Zhang R 2013	✓	✓	✓		✓			✓	✓	**6**
Zhang Y 2015	✓	✓	✓	✓	✓	✓		✓	✓	**8**
Zhang Y 2017	✓	✓	✓	✓	✓	✓		✓	✓	**8**
Zhang Y 2020	✓	✓	✓	✓	✓			✓	✓	**7**
Zhou HX 2016	✓		✓	✓	✓			✓	✓	**6**

Supplementary Table 3: Risk of Bias (RoB)

Legend: low risk; unclear risk; high risk

Author/year	selection bias			performance bias		detection bias		attrition bias	reporting bias	other bias
	sequence generation	baseline characteristics	allocation concealment	random housing	blinding (study team)	random outcome assessment	blinding (outcome assessment)	incomplete outcome data	selective outcome reporting	free influence of funders
Anbari F 2014	unclear	low	unclear	unclear	unclear	unclear	unclear	low	low	low
Bakhtiary M 2010	unclear	low	unclear	unclear	low	unclear	low	low	low	unclear
Bakhtiary M 2011	unclear	unclear	unclear	unclear	low	unclear	low	low	low	unclear
Bonilla C 2009	unclear	low	unclear	unclear	unclear	high	unclear	unclear	unclear	low
Bonilla C 2012a	unclear	low	unclear	unclear	unclear	unclear	unclear	low	low	low
Bonilla C 2012b	unclear	low	unclear	unclear	unclear	unclear	unclear	low	low	low
Bonilla C 2014	unclear	low	unclear	unclear	unclear	unclear	unclear	low	low	low
Bonilla C 2018	unclear	low	unclear	unclear	low	unclear	low	low	low	low
Chang CP 2013	unclear	high	unclear	unclear	low	unclear	low	low	low	unclear
Chen L 2014	unclear	low	unclear	unclear	unclear	unclear	unclear	low	low	unclear
Cheng T 2015	unclear	low	unclear	unclear	low	unclear	low	high	high	low
Danilina TI 2017	unclear	low	unclear	unclear	unclear	unclear	unclear	low	low	unclear
Darkazalli A 2016	unclear	high	unclear	unclear	unclear	unclear	unclear	low	low	unclear
Guan F 2019	unclear	unclear	unclear	unclear	low	unclear	low	low	low	low
Guan J 2013	unclear	low	unclear	unclear	low	unclear	low	low	low	unclear
Han EY 2013	unclear	low	unclear	unclear	low	unclear	low	low	low	low
Harting MT 2009	low	low	unclear	unclear	low	high	low	unclear	unclear	low
Hong SQ 2011	unclear	high	unclear	unclear	unclear	unclear	unclear	low	low	unclear
Hu H 2020	unclear	low	unclear	unclear	low	high	low	low	high	low
Hu J 2019	unclear	high	unclear	unclear	low	unclear	low	low	low	low
Hu W 2018	unclear	high	unclear	unclear	low	unclear	low	high	low	low
Jiang Q 2011	unclear	low	unclear	unclear	low	unclear	low	low	low	unclear
Kappy NS 2018	unclear	low	unclear	unclear	unclear	unclear	unclear	low	low	low
Kim C 2018	unclear	unclear	unclear	unclear	unclear	unclear	unclear	low	low	unclear
Kim DK 2016	unclear	high	unclear	unclear	unclear	unclear	unclear	low	low	low
Kim HJ 2010	unclear	low	unclear	unclear	unclear	unclear	unclear	unclear	low	low
Kota DJ 2015	unclear	high	unclear	unclear	low	unclear	low	low	low	low
Lam PK 2016	unclear	unclear	unclear	unclear	unclear	unclear	unclear	unclear	low	low
Li L 2011	unclear	low	unclear	unclear	low	unclear	low	low	low	low
Li L 2012	unclear	unclear	unclear	unclear	low	low	low	low	low	low
Li L 2017	unclear	unclear	unclear	unclear	low	unclear	low	low	high	low
Li L 2017	low	high	unclear	unclear	unclear	unclear	unclear	unclear	low	low
Lin CH 2019	unclear	low	unclear	low	high	unclear	high	low	low	low
Liu Y 2014	unclear	low	unclear	unclear	unclear	unclear	unclear	low	low	unclear
Lu D 2001	low	low	unclear	unclear	low	high	low	high	high	unclear
Lu D 2002	low	low	unclear	unclear	unclear	high	low	low	low	high
Lu D 2007	unclear	low	unclear	unclear	low	high	unclear	unclear	unclear	unclear
Lu M 2003	unclear	low	unclear	unclear	unclear	high	low	low	low	unclear
Ma J 2015	unclear	high	unclear	unclear	low	unclear	low	high	high	low
Mahmood A 2001	low	unclear	unclear	unclear	low	high	low	low	low	unclear
Mahmood A 2002	low	low	unclear	unclear	low	high	low	low	low	unclear
Mahmood A 2003	low	low	unclear	unclear	low	high	low	high	low	unclear
Mahmood A 2004b	low	unclear	unclear	unclear	low	high	low	low	low	unclear
Mahmood A 2005	low	unclear	unclear	unclear	low	high	low	low	low	unclear
Mahmood A 2006	low	low	unclear	unclear	low	high	low	unclear	unclear	unclear
Mahmood A 2007	unclear	low	unclear	unclear	low	high	unclear	unclear	unclear	unclear
Mahmood A 2008	low	low	unclear	unclear	low	high	low	unclear	unclear	unclear
Mahmood A 2013	unclear	low	unclear	unclear	low	unclear	low	low	low	low
Maruichi K 2009	unclear	low	unclear	unclear	unclear	unclear	unclear	low	low	unclear
Mastro-Martinez I 2015	unclear	high	unclear	unclear	low	unclear	low	low	low	low
Mishra SK 2017	unclear	high	unclear	unclear	unclear	unclear	unclear	low	low	low
Nichols JE 2013	unclear	high	unclear	unclear	unclear	low	unclear	low	low	low
Patel NA 2018	unclear	unclear	unclear	unclear	low	unclear	low	unclear	unclear	low

Legend:
- low risk
- unclear risk
- high risk

Author/year	selection bias: sequence generation	selection bias: baseline characteristics	selection bias: allocation concealment	performance bias: random housing	performance bias: blinding (study team)	detection bias: random outcome assessment	detection bias: blinding (outcome assessment)	attrition bias: incomplete outcome data	reporting bias: selective outcome reporting	other bias: free influence of funders
Peruzzaro ST 2019	unclear	high	unclear	unclear	unclear	unclear	unclear	high	unclear	low
Pischiutta F 2014	unclear	high	low	unclear	low	unclear	low	low	low	low
Pischiutta F 2016	low	unclear	low	unclear	low	unclear	low	low	low	low
Qu C 2008	unclear	high	unclear	unclear	low	high	unclear	unclear	unclear	unclear
Qu C 2009	unclear	high	unclear	unclear	unclear	unclear	unclear	low	low	unclear
Qu C 2011	unclear	low	unclear	unclear	low	unclear	low	low	low	unclear
Ruppert KA 2020	unclear	low	unclear	unclear	low	unclear	low	high	low	high
Sharor RA 2020	unclear	high	unclear	unclear	unclear	unclear	unclear	low	low	low
Shi X 2018a	unclear	high	unclear	unclear	unclear	unclear	unclear	low	low	low
Shi X 2018b	unclear	high	unclear	unclear	unclear	unclear	unclear	low	low	low
Shin MS 2016	unclear	high	unclear	unclear	unclear	unclear	unclear	low	low	low
Silachev DN 2015	low	low	unclear	unclear	unclear	unclear	unclear	low	low	unclear
Tajiri 2014	unclear	high	unclear	unclear	low	unclear	low	low	low	low
Turtzo LC 2015	low	low	unclear	unclear	unclear	unclear	unclear	low	low	low
Wang S 2013	unclear	low	unclear	unclear	low	unclear	low	low	high	low
Wang Z 2013	unclear	high	unclear	unclear	low	unclear	low	low	low	unclear
Wang Z 2015	unclear	high	unclear	unclear	unclear	unclear	unclear	low	low	low
Wu K 2019	unclear	low	unclear	unclear	low	unclear	low	low	low	low
Xiong Y 2009	low	low	unclear	unclear	low	high	low	unclear	unclear	unclear
Xu H 2020	unclear	low	unclear	unclear	unclear	unclear	unclear	low	low	low
Xu HS 2014	unclear	low	unclear	unclear	unclear	unclear	unclear	low	low	low
Xu L 2019	unclear	low	unclear	unclear	low	unclear	low	high	high	low
Yan F 2019	unclear	low	unclear	unclear	unclear	unclear	unclear	high	low	low
Yan ZJ 2013	unclear	low	unclear	unclear	unclear	unclear	unclear	low	low	low
Yao M 2019	unclear	high	unclear	unclear	low	unclear	low	low	low	low
Yuan X 2020	unclear	low	unclear	unclear	low	unclear	low	low	low	low
Yuan Y 2014	unclear	high	unclear	unclear	low	unclear	low	high	high	low
Zanier ER 2011	low	low	low	high	low	high	low	low	low	low
Zanier ER 2014	low	unclear	low	high	low	unclear	low	low	low	low
Zhang J 2012	unclear	high	unclear	unclear	low	unclear	low	high	high	low
Zhang R 2013	low	unclear	unclear	unclear	unclear	unclear	unclear	low	high	low
Zhang Y 2015	low	low	low	unclear	low	unclear	low	low	low	low
Zhang Y 2017	unclear	low	low	unclear	low	unclear	low	low	low	low
Zhang Y 2020	unclear	low	unclear	unclear	low	unclear	low	low	low	low
Zhou HX 2016	unclear	low	unclear	unclear	low	unclear	low	low	low	low

Supplementary Table 4: SMD and statistics

Neurologic assessment				
	number of comparisons	SMD [95% CI]	*P* value	I^2
**Naive MSC**				
baseline	38	-0.04 [-0.20, 0.11]	0.590	0.00%
1 week	45	-1.09 [-1.39, -0.80]	<0.0001	65.58%
2 weeks	42	-1.34 [-1.64, -1.04]	<0.0001	66.98%
3 weeks	42	-1.92 [-2.48, -1.36]	<0.0001	79.00%
4 weeks	24	-1.68 [-2.11, -1.24]	<0.0001	81.35%
5 weeks	12	-1.17 [-1.50, -0.84]	<0.0001	13.23%
last time point	57	-1.54 [-1.85, -1.23]	<0.0001	74.27%
**Labeled**				
baseline	7	-0.20 [-0.63, 0.23]	0.364	0.00%
1 week	11	-1.35 [-1.96, -0.74]	<0.0001	60.38%
2 weeks	11	-2.07 [-3.21, -0.93]	0.0004	86.08%
3 weeks	4	-2.67 [-3.69, -1.66]	<0.0001	56.67%
4 weeks	5	-1.72 [-2.33, -1.11]	<0.0001	28.14%
5 weeks	4	-2.47 [-3.92, -1.11]	0.0008	80.03%
last time point	12	-2.01 [-2.92, 1-11]	<0.0001	80.54%
**Naive+Labeled**				
baseline	45	-0.06 [-0.21, 0.09]	0.410	0.00%
1 week	56	-1.14 [-1.41, -0.88]	<0.0001	64.72%
2 weeks	53	-1.46 [-1.77, -1.15]	<0.0001	73.16%
3 weeks	46	-2.02 [-2.53, -1.52]	<0.0001	77.70%
4 weeks	29	-1.66 [-2.04, -1.29]	<0.0001	78.06%
5 weeks	16	-1.39 [-1.76, -1.01]	<0.0001	44.79%
last time point	69	-1.61 [-1.90, -1.32]	<0.0001	74.54%

Hindlimb function				
	number of comparisons	SMD [95% CI]	*P* value	I^2
**Naive+Labeled**				
baseline	6	-0.20 [-0.59, 0.18]	0.299	0.00%
1 week	10	-0.93 [-1.59, -0.27]	0.006	73.29%
2 weeks	8	-0.92 [-1.61,-0.23]	0.009	68.65%
3 weeks	8	-1.61 [-2.00, -1.22]	<0.0001	0.00%
4 weeks	7	-1.68 [-2.26, -1.10]	<0.0001	44.41%
5 weeks	6	-1.45 [-1.91, -0.99]	<0.0001	0.00%
last time point	12	-1.43 [-2.05, -0.81]	<0.0001	71.53%

Coordination				
	number of comparisons	SMD [95% CI]	*P* value	I^2
**Naive+Labeled**				
baseline	13	0.06 [-0.21, 0.32]	0.676	0.00%
1 week	15	0.75 [0.20, 1.30]	0.008	67.14%
2 weeks	16	1.40 [0.57, 2.24]	0.001	84.55%
3 weeks	5	1.02 [0.44, 1.59]	0.001	0.00%
4 weeks	11	0.99 [0.31, 1.67]	0.004	75.97%
5 weeks	4	0.41 [-0.53, 1.35]	0.391	40.10%
last time point	22	1.21 [0.70, 1.71]	<0.0001	73.99%

Stratified meta-analysis, neurologic assessment, last time point				
	**number of comparisons**	**SMD [95% CI]**	***P* value for subgroup differences**	**I^2**
Pooled estimate (naive+labeled)	69	-1.61 [-1.89, -1.32]	<0.0001	74.54%
**Source**				
BM	53	-1.55 [-1.86, -1.24]	<0.0001	70.95%
UC	8	-1.69 [-2.72, -0.66]	0.001	85.20%
UCB	3	-2.55 [-4.35, -0.75]	0.006	81.95%
PL	3	-2.68 [-4.61, -0.76]	0.006	81.38%
AD	2	-0.26 [-1.22, 0.70]	0.600	19.06%
**Immunogenicity**				
syngenic	30	-1.25 [-1.66, -0.84]	<0.0001	71.89%
xenogenic	38	-1.84 [-2.22, -1.45]	<0.0001	72.71%
**Time of transplant**				
2-12 h	5	-1.78 [-2.39, -1.18]	<0.0001	19.14%
24h	38	-1.77 [-2.19, -1.34]	<0.0001	77.58%
>1d to 1week	19	-1.69 [-2.28, -1.11]	<0.0001	74.52%
>1 week	7	-0.67 [-1.26, -0.08]	0.026	61.57%
**Route of administration**				
IV	42	-1.42 [-1.78, -1.05]	<0.0001	73.58%
cavity	12	-2.54 [-3.48, -1.60]	<0.0001	82.33%
IC	7	-1.80 [-2.76, - 0.84]	0.0002	71.67%
ICV	7	-1.54 [-1.98, -1.10]	<0.0001	26.93%
**Amount (systemic)**				
≤1.5M	10	-0.62 [-0.98, -0.26]	0.001	0.00%
2M	13	-1.95 [-2.70, -1.20]	<0.0001	73.62%
3M	5	-2.46 [-3.48, -1.44]	<0.0001	61.70%
4M	6	-2.03 [-3.75, -0.30]	0.022	88.41%
>8M	4	-0.48 [-0.91, -0.04]	0.032	0.00%
**Amount (cerebral)**				
≤1M	7	-1.38 [-1.88, -0.88]	<0.0001	28.44%
2M	3	-4.27 [-9.28, 0.74]	0.095	94.11%
3M	5	-2.43 [-3.23, -1.63]	<0.0001	25.24%
5M	4	-1.20 [-1.67, -0.74]	<0.0001	0.00%
**Species**				
rat	59	-1.66 [-2.01, -1.31]	<0.0001	78.65%
mouse	10	-1.43 [-1.77, -1.08]	<0.0001	11.18%
**Sex**				
male	44	-1.92 [-2.29, -1.55]	<0.0001	73.10%
female	17	-0.91 [-1.30, -0.52]	<0.0001	56.83%
unclear	8	-1.35 [-2.20, -0.50]	0.002	70.46%
**TBI model**				
CCI	44	-1.59 [-1.93, -1.25]	<0.0001	69.29%
WD	19	-1.91 [-2.64, -1.19]	<0.0001	85.75%
FPI	6	-1.11 [-1.97, -0.25]	0.011	73.20%

MSC modifications, neurologic assessment, last time point				
	**number of comparisons**	**SMD [95% CI]**	***P* value**	**I^2**
Naive+Labeled	69	-1.61 [-1.90, -1.32]	<0.0001	74.54%
Matrix embedded	11	-7.73 [-11.31; -4.15]	<0.0001	95.84%
Genetically modified	5	-4.24 [-6.99, -1.48]	<0.01	94.43%
MSC derivatives	11	-4.32 [-5.94, -2.70]	<0.0001	83.81%

Cognitive function				
	number of comparisons	SMD [95% CI]	*P* value	I^2
**Naive+Labeled MSCs**				
1+2+3 week	20	0.91 [0.49, 1.33]	<0.0001	59.49%
4+5 weeks	23	2.11 [1.56, 2.66]	<0.0001	76.32%
**Matrix embedded MSCs**				
4+5 weeks	9	4.60 [3.24, 5.97]	<0.0001	79.15%
**MSC-derivatives**				
4+5 weeks	12	2.91 [1.88, 3.94]	<0.0001	76.96%

Contusion volume				
	number of comparisons	SMD [95% CI]	*P* value	I^2
**Naive+Labeled MSCs**				
1+2 week	14	0.24 [-0.04, 0.51]	0.092	0.00%
4+5 weeks	20	1.34 [0.89, 1.80]	<0.0001	66.68%
**Matrix embedded MSCs**				
4+5 weeks	3	2.25 [1.64, 2.87]	<0.0001	0.00%
**Genetically modified MSCs**				
4+5 weeks	3	2.56 [1.28, 3.84]	<0.0001	35.33%
**MSC-derivatives**				
4+5 weeks	3	0.63 [-0.03, 1.30]	0.060	0.00%

Supplementary Table 5: Key search terms used in database searches

TBI	MSC
brain injuries	mesenchymal stromal cell
traumatic brain injury	mesenchymal stem cell
traumatic brain injuries	mesenchymal stromal cells
brain contusion	mesenchymal stem cells
brain trauma	marrow stromal cell
head trauma	bone marrow stem cell
traumatically injured brain	bone marrow derived stromal cell
diffuse axonal injury	Wharton's jelly
head trauma	umbilical cord stroma
diffuse axonal injuries	stromal vascular fraction stem cell
head injuries	menstrual stem cell

Queries used with Boolean operators in PUBMED and EMBASE databases

PUBMED

("Brain Injuries"[Mesh] OR "traumatic brain injury" OR "traumatic brain injuries" OR "brain contusion" OR "brain trauma" OR “head trauma” OR "traumatically injured brain" OR "diffuse axonal injury" OR "diffuse axonal injuries" OR “head injury” OR “head injuries”)
AND
((((((((((((((((((((((((((((((((((((((("Mesenchymal Stromal Cells"[Mesh]) OR mesenchymal OR "mesenchymal stem cell") OR "mesenchymal stem cells") OR "mesenchymal stromal cell") OR "mesenchymal stromal cells") OR "marrow stromal cell") OR "marrow stromal cells") OR "bone marrow stem cell") OR "bone marrow stem cells") OR "bone marrow derived stromal cell") OR "bone marrow derived stromal cells") OR "mesenchymal precursor cell") OR "mesenchymal precursor cells") OR MSC[Title/Abstract]) OR MSCs[Title/Abstract]) OR BMSC[Title/Abstract]) OR BMSCs[Title/Abstract]) OR "Wharton Jelly"[Mesh]) OR "wharton's jelly") OR "wharton jelly") OR "umbilical cord stroma") OR "umbilical cord") OR "Fetal Blood"[Mesh]) OR "umbilical cord blood") OR UCB[Title/Abstract]) OR "adipose-derived stromal cell") OR "adipose-derived stromal cells") OR ASC[Title/Abstract]) OR ASCs[Title/Abstract]) OR "adipose stem cell") OR "adipose stem cells") OR "pre-adipocyte") OR "pre-adipocytes") OR "stromal vascular fraction stem cell") OR "stromal vascular fraction stem cells") OR "adipose tissue derived stem cell") OR "adipose tissue derived stem cells") OR "dental pulp") OR "menstrual stem cell")

EMBASE

(‘Traumatic brain injury'/exp OR 'traumatic brain injury' OR 'brain contusion' OR 'head injury' OR 'diffuse axonal injury') AND ('mesenchymal stem cell'/exp OR 'mesenchymal stroma cell'/exp OR 'mesenchymal stem cell' OR 'bone marrow stroma cell' OR 'mesenchymal precursor cells' OR 'wharton jelly' OR 'umbilical cord' OR 'adipose derived stem cell' OR 'menstrual stem cells')
